# Supplementary material for: Evidence suggesting that reindeer mothers allonurse according to the direct reciprocity and generalized reciprocity decision rules
Source: PLoS One. 2023 Dec 14;18(12):e0295497. doi: 10.1371/journal.pone.0295497 (PMC10721201; doi:10.1371/journal.pone.0295497)
Supplement: Attachment — Submitted filename: PONE_D_22_28248.pdf [file pone.0295497.s012.pdf]

Overview of comments:

The authors have attempted to study three hypotheses to understand allonursing in semi-domesticated reindeer: direct reciprocity, generalized reciprocity, and kin selection. The authors have also attempted to compare direct and generalized reciprocity in the study population, which seemingly present interesting results, as well as the interaction between direct reciprocity and kin selection.

The authors have cited relevant literature for the hypotheses but have not discussed them adequately. Apart from discussing various hypotheses for allonursing, it will be useful to also briefly discuss the various factors – such as mother's age, offspring sex, offspring age, etc., that are known to affect allonursing in wild and/or domesticated reindeer populations. It is necessary to describe the study species' social organization and structure and how it differs from the study population, as allonursing should be understood in the context of the species' biology.

Unfortunately, the study suffers from severe methodological problems. The data on allonursing bouts were collected over a span of 10 and 5 weeks in 2012 and 2013 following two different methods. Overall, I do not think it is appropriate to pool 2012 and 2013 data during analysis. Nor should that data on two groups be pooled for 2013; each group should be a data point. Though it is theoretically possible to use a nested model (as each individual appears in only one year), the method of data collection is very different between 2012 and 2013, so it will not be appropriate to pool data across years or groups. This, I believe, is one of the major problems with the study.

The best that can be done is to analyse 2012 data separately and 2013 data separately, however, this will require different methods of statistical analysis. I would even recommend the authors to use only the 2012 data and analyse them only for direct and generalized reciprocity, as the predictor range is very narrow for genetic relatedness. However, the 2012 and 2013 data are already published.

The methods, primarily, and the results section will have to be written in much more detail to make it possible for anyone to understand and replicate the study. The conclusions are drawn from inappropriate data analysis and through extrapolating the results, lead to the conclusions not being supported by the data. Specific recommendations are written below (in blue).

The manuscript in its current form is not suitable for publication due to major problems with data collection protocol including artificially constructing groups that can easily lead to erroneous results, insufficient data to test functional hypotheses, and improper analyses on previously published data. Unfortunately, the manuscript will have to be rejected.

Detailed comments:

1. "Altruism, i.e. a behaviour costly to the actor and beneficial to the recipient (1), was one of Darwin's greatest challenges to his theory of natural selection."

Since there is a vast body of work on altruism subsequently, starting the manuscript with an allusion to Darwin is like setting up a straw-man.

2. “Reciprocity is an apparently helpful behaviour,...”

Why is it apparently helpful as opposed to helpful?

3. “Cooperation was better explained by direct reciprocity than relatedness (22,24,48) and generalized reciprocity (38) in empirical studies.”

There are a lot of studies of cooperation with evidence for both relatedness and reciprocity.

The introduction should be shortened and follow a logical flow. Also, please remove details of various hypotheses that are not being tested in the paper. They can just be listed.

4. “Relatedness did not influence the odds of allonursing (73), however a group of closely-related mothers allonursed each other’s offspring more often than a group of distantly-related mothers at the extremes of pairwise genetic relatedness (72).”

Given this, why is genetic discrimination being tested again in this paper?

5. “Although we previously found partial support for reciprocal allonursing in reindeer (70), we need to assess i) the evolved, mechanistic decision rules of direct reciprocity, generalized reciprocity and kin discrimination responsible for cooperation, and ii) the interaction between the direct reciprocity and kin discrimination decision rules.”

Why this needs to be assessed should be the crux of the introduction.

6. “In 2012, the first 25 offspring born and their mothers were selected and studied for 10 weeks.”

What was the reason for choosing only the first 25 offspring?

7. “In 2013, we obtained 2 groups and each group consisted of 8 mother-offspring pairs based on their pairwise genetic relatedness, forming 2 groups for 5 weeks at the extremes of pairwise genetic relatedness: one group of closely, genetically related mothers and one group of distantly, genetically related mothers.”

From reading the methods section of this manuscript and those of the cited works from the research group (Engelhardt *et al*. 2014, 2015, 2016 a,b or references 70-73), I understand that the methods followed in 2012 and 2013 are different.

In 2012, 25 mother-offspring pairs were separated from the larger herd, and behavioural observations were taken on them. In 2013, a group each of closely and distantly related mothers (along with their offspring) were formed, and behavioural observations were taken separately on these groups.

What was the necessity to form two separate groups in 2013? If it is to check for the effect of relatedness, forming two separate groups is not necessary. Relatedness can be treated as a continuous variable and its effect on the number of help given or received can be analysed.

Ultimately, the researchers have pooled all the data from 2012 and 2013 to analyse the effect of direct and generalized reciprocity and kin discrimination. This again brings up the question: why form two groups at all in 2013?

Please provide the average ± 95%CI as well as range of pairwise genetic relatedness for both the years as well as groups.

Year was included as a random factor in only two of the four models run. The effect of year was not included in the results. I suggest the authors to include the effect of all the fixed and random factors included in all the models in the results section.

The period of study between the two years is also different (2012: 10 weeks, 2013: 5 weeks). This would mean that calves born in 2012 would have been followed for a longer duration of their infancy than the calves born in 2013. If I understand correctly, the ages of offspring of the two groups of 2013 were similar but not the ages of offspring of 2012 and 2013. Please provide justifications for how the difference in the length of infancy period covered would not affect the results. As far as I understand, age of offspring was not included as a factor in any of the models.

8. "The study animals were separated from the herd until the end of the study, after which they joined the rest of the herd."

What was the reason for separating the study animals from the larger herd? Would it not artificially restrict who was available for allonursing and bias the results?

Would it also not influence the natural behaviour of the semi-domesticated animals? I understand that comparing the behaviours of mothers before and after separation from the larger herd would not be possible due to the study design. However, were the behaviours of separated mothers compared with that of mothers who were not separated? Were the behaviours of mothers compared after separation and after rejoining? If the authors can provide justifications for separation not affecting individual behaviour, it would be helpful.

Separating the females from the herd for study will also introduce confounding factors. The factors influencing allonursing are likely to be highly dependent on evolved social systems. Therefore, the study should be situated within social groupings that the females are used to. Inferences made by separating females from the original social grouping may not hold at all in a more natural scenario. Much more detail should be provided about the social structure of reindeer.

Out of curiosity: Does pairwise familiarity, and not relatedness, affect allonursing in the study population? Is it difficult to tease apart the effect of familiarity vs relatedness?

9. "A successful allonursing bout was considered terminated when an offspring's muzzle and a lactating female's udder were not in contact for 20 s or more."

How was the 20 s cut-off decided? Please state the reasons.

Though successful and unsuccessful allonursing bouts are differentiated, how are independent and non-independent allonursing bouts differentiated? If independent data points are not selected for the analyses, one would run into autocorrelation (pseudoreplication) issue.

10. “Reindeer nursing bouts last 2.7 times longer than allonursing bouts (nursing: 41 s; allonursing: 15 s)”

Please specify the average durations of nursing and allonursing bouts along with 95% CI and sample sizes.

Out of curiosity: If the duration of allonursing was analysed with respect to the degree of pairwise genetic relatedness between the mothers, would the results of this paper have been different? Is it possible for mothers to allonurse to similar frequencies regardless of their relatedness, but allonurse to differing durations with respect to their relatedness?

Regarding relatedness: Is there any evidence to suggest that reindeers identify their maternal and/or paternal kin either in the wild or in captivity? Even if there isn’t any evidence, I suggest that the authors include a couple of sentences about kin recognition in the introduction when introducing the kin discrimination hypothesis.

11. “In 2012, all reindeer mothers allonursed 1 or more offspring, and we recorded 1383 allonursing bouts (73). In the closely and distantly, genetically related groups in 2013, we recorded 113 and 48 allonursing bouts, respectively, performed by 8 of the 8 reindeer mothers in the closely-related group and by 7 of the 8 reindeer mothers in the distantly-related groups (72).”

Please provide the average ± 95%CI of the frequency of nursing and allonursing bouts for 2012 and for both the groups of 2013.

1. What was the focal duration for each experimental mother dyad?
2. Was the focal duration held constant across all the experimental dyads within and between years? I do not think so (at least for between years): this is evident from the huge difference in the frequency of allonursing bouts between 2012 and 2013.
3. If not, did the authors correct for inter-dyad variation in focal duration when calculating frequencies of help given and help received?

Please include these in the manuscript (perhaps as a supplementary information).

How many researchers coded the behavioural data? If this was more than one, was there high inter-observer reliability? How was it measured? Please include this in the methods.

12. “In this study, we revisited the associations between help given and i) help received and ii) pairwise genetic relatedness and their interactions by updating the assessment of reciprocal and kin discriminated allonursing by including all data collected in 2012, i.e. all allonursing bouts lasting > 5 s rather than only the data with exact known duration, and by adding a 2nd year of data, i.e. data collected in 2013.”

This takes me back to the previous-to-previous comment. Have the researchers checked for the effect of relatedness on allonursing duration? It is possible for frequencies and durations to produce different results. Without this knowledge, it is inappropriate to include all the bouts >5 s without weighting them in the models. I suggest the authors to first make sure that frequencies and durations of allonursing bouts follow similar pattern with respect to pairwise relatedness and other predictors. Alternatively, perform two sets of analyses: one with the

frequencies and the other with the durations of allonursing bouts and compare the results. Just based on the results of frequencies one cannot conclude the effect of relatedness. Or the conclusion should be such that pariwise genetic relatedness does/does not significantly influence frequency of allonursing in this population of semi-domesticated reindeers.

13. "We generated a dominance hierarchy for reindeer mothers in each group using observations of agonistic interactions with a winner and loser identified. We reported the Landau linearity index (86). The dominance hierarchy in the closely related group tended to be linear, with a Landau linearity index of 0.857 (72). The dominance hierarchy in the distantly related group was linear, with a Landau linearity index of 0.988 (72). In 2012, the dominance hierarchy tended to be linear, with a Landau's index of linearity of 0.785 (70). Ranks were given values ranging 1 to 25 in 2012 and ranging 1 to 8 in both groups in 2013, with larger numbers representing the most dominant mother and 1 representing the least dominant mother."

Please mention the behaviours that were used to score agonistic interactions (which behaviours, descriptions of behaviours, their frequency, how winners and losers were identified, etc.). If this has been already described in any of the published papers, include references. Please also include the duration of observation for each dyad during which the agonistic interactions were scored. Was this similar across dyads or was it controlled for before constructing the dominance hierarchies?

In 2012, a dominance hierarchy was constructed with all the study individuals regardless of their relatedness. However, in 2013, two separate dominance hierarchies were constructed based on relatedness (i.e., for the two groups). Therefore, the highest absolute rank difference can be 24 in 2012 but only 7 in 2013, which can be also seen in the file Data 1.csv. Is there any evidence to support that these two methods of constructing dominance hierarchies with respect to relatedness would not affect the results? The replicates for rank differences ranging from 8 to 24 will only be from the year 2012, but the replicates for the rank differences ranging from 1-7 will be from both 2012 and 2013. Please bear in mind that these replicates (2012 and 2013) are not similar because of the major difference in the method of constructing the hierarchies.

1. If certain individuals are removed from the larger herd and then the dominance hierarchy is constructed, does the rank relationship between pairs of females remain the same as when they were in the larger herd?
2. If it does not, what do the (temporary) absolute rank differences in the experimental groups mean for the individuals?
3. Do the rank differences in the experimental groups have any biological significance at all?
4. How stable are these dominance ranks? I would think it will take more than the experimental period for the ranks of individuals to become stable, unless, for e.g., if A>B>C is the case and upon removing B, the relationship is still A>C immediately from the time B is removed and continues to be the case if B does not return. If the dominance hierarchy is constructed for the entire herd, would the researchers find this to be the case? Essentially, though the ranks of the experimental animals would themselves differ upon removal from the larger herd, would the hierarchical positions of the experimental animals remain the same before and after removal?
5. Please provide information on when and for how long the dominance data were collected in each year.

14. “To assess the direct reciprocity decision rule and the interaction between the direct reciprocity and kin discrimination decision rules, we ran a generalized linear mixed model with a Poisson distribution with the number of help given as a response variable, and the interaction between the number of help received and the pairwise genetic relatedness, the absolute rank difference, the offspring birth mass difference, the similarity in offspring sex (categorical variable) as fixed effects.”

From R script: m1_givereceive_data_int <-glmer(givencount ~ receivedcount*LRM + ABSOLUTERANKDIFFERENCE + sexsimilarity + birthmassdiff + (1 | id1_factor) + (1 | id2_factor) + (1|year), family="poisson", data=givereceive_data)
summary(m1_givereceive_data_int) # interaction is non-sig
overdisp_fun(m1_givereceive_data_int)
vif(m1_givereceive_data_int)

m1_givereceive_data <-glmer(givencount ~ receivedcount + LRM + ABSOLUTERANKDIFFERENCE + sexsimilarity + birthmassdiff + (1 | id1_factor) + (1 | id2_factor) + (1|year), family="poisson", data=givereceive_data)
#,glmerControl(optimizer="bobyqa", optCtrl = list(maxfun = 100000))
summary(m1_givereceive_data)
overdisp_fun(m1_givereceive_data)
vif(m1_givereceive_data)

“The interaction between the number of help received and pairwise genetic relatedness did not significantly affect the number of help given ($\beta \pm SE$: 0.25 ± 0.21, P = 0.24), which did not support our prediction of the interaction between the direct reciprocity and kin discrimination decision rules: the interaction was removed from the model.”

Please state how ‘sexsimilarity’ was coded in the model. Was it something similar to FF = -1, FM = 0, MF = 1, MM = 2 or FF = 0 and MM = 1?

What about the factors offspring age difference as well as mothers’ age difference for each dyad? Do they bear any effect on help given? Is there collinearity between female age and female rank? If so, checking age differences between mothers would not be necessary. Otherwise, consider adding these factors to the model. Later run reduced models and perform likelihood ratio tests with all the models to choose the best model/s.

Here, it is the dyad on which the data on help given and received are scored rather than on individual mothers as in the case of generalized reciprocity. Therefore, here dyad identity should be the random factor (as dyads are the replicates) and not individual mothers of the dyad. (i.e., (1 | id1_id2) instead of (1 | id1_factor) + (1 | id2_factor)).

A link function has not been included in the code. For Poisson models, the link function should be log. Please check if R takes link function as log for Poisson models by default. If not, the models will have to be rerun with the appropriate link function.

The model should not be run only with the factor receivedcount*LRM, and the interaction should not be removed just because the *P* value is not significant. The original hypothesis was to check for the effect of the interaction term; therefore, it should be retained regardless of its *P* value. Full model has to be run, and the inferences will have to be made based on the results of the best model. The model should use receivedcount + LRM + receivedcount*LRM.

In the methods section, please write in detail about the model diagnostics that were run – for e.g.,

1. How was independence of residuals tested?
2. How was homogeneity of variance tested?
3. How was model stability tested (DFBeta/DFFit)? If the model is unstable, this reduces inferential power, and the conclusions should be drawn with care.

15. "There were 5 outliers for the number of help received, 2 outliers for pairwise genetic relatedness, and 1 outlier for offspring birth mass difference with an absolute z-score values greater than 3.3. The outliers were real data points, so we kept them. To assess the impact of these outliers, we changed them to the highest value below an absolute z-score of 3.3, and we ran the model with these corrected outlier values."

How was this done? Which function was used? Please write these clearly in the methods sections and provide references for this method. Without going through the data and the R code, it will be impossible for anyone to understand or replicate this step. DFBeta (mentioned above) is a good measure to detect influential cases.

16. "Observations of successful allonursing across bouts were summed within dyads for the closely and distantly, genetically related groups. To assess dyadic reciprocity, we used the reciprocity index presented by Mitani (84), which has been used to assess dyadic reciprocity for allogrooming (84,85) and for allonursing (70), to create the reciprocal allonursing frequency index (RAFI).

$$RAFI = 1 - \left|\frac{aAB}{aA+aB} - \frac{aBA}{aA+aB}\right| \quad (1)$$

Where *aAB* is the amount of allonursing that individual *A* gave to individual *B*'s offspring, *aBA* is the amount of allonursing that individual *B* gave to individual *A*'s offspring, and *aA* + *aB* is the total amount of allonursing between the two individuals."

If I have understood this correctly, only the successful allonursing bouts were considered to calculate RAFI. It is then not meaningful to run the below model:

17. "To further assess the direct reciprocity decision rule and the interaction between the direct reciprocity and the kin discrimination decision rules, we ran a generalized linear mixed model with a binomial distribution with successful (1) and unsuccessful (0) allonursing attempts as a response variable, and RAFI, pairwise genetic relatedness, offspring sex (categorical variable), and absolute rank difference. The response variable of the likelihood of allonursing model equals 0 when an attempt is unsuccessful, i.e., the lactating female did not allonurse the offspring of another mother, and equals 1 when an attempt is successful, i.e., the lactating female did allonurse the offspring of another mother."

This is because higher the RAFI higher would be the likelihood of successful allonursing.

1. It would be better to design this model as:

   glmer (RAFI ~ pairwise genetic relatedness + absolute rank difference + similarity in offspring sex + id1_id2 age difference + offspring1_offspring2 age difference +

offspring1_offspring2 birth mass difference + (1 | id1_id2) + (1 | year), family = “X”, link = “x”, data = data)

through which it is possible to understand if genetic relatedness, rank difference, etc., has any effect on the extent of direct reciprocity. Here again reduced models would have to be run and the full and the reduced models should be compared with likelihood ratio tests. Variance Inflation Factor should also be measured for the model.

Here again, upon going through the R script, dyad identity should be the random factor and not the identities of the individual mothers.

2. Alternatively, Mantel tests can be performed with the pairwise RAFI and genetic relatedness matrices separately for each year.

18. “For each model, when the interaction between pairwise genetic relatedness and either i) the number of help received or ii) RAFI was not significant, the interaction was removed from the model.”

As explained above, individual factors should not be removed post hoc based on *P* value. Complete models will have to be run unless there is a strong reason to not do so.

“To assess the generalized reciprocity decision rule, we asked if receiving help in general increased subsequent help given, and we ran a general linear model with a negative binomial distribution with the overall number of help given by each reindeer mother as a response variable, and the overall number of help received by each reindeer mother as an independent variable.”

From R script: m2_givereceive_gen_rep <-glm.nb(givencount ~ receivedcount, data=givereceive_gen_recip)

1. Please state the reason for using a negative binomial distribution (overdispersion); include the function used and the reference.
2. It is important to add mother_ID (as there are repeated observations on the same individuals) and year as random factors (if same method was followed for both the years) – i.e., (1 | mother_ID), (1 | year).
3. Factors such as mother’s age, mother’s dominance rank, offspring sex, relatedness to the receiver should be included as control fixed factors, and VIF should be measured.
4. If the focal duration is not the same across different mothers, please include offset.

19. “To compare direct reciprocity and generalized reciprocity, we ran a generalized linear mixed model with a Gaussian distribution with the latency to give help after receiving help in days as a response variable, the comparison between the direct reciprocity and generalized reciprocity decision rules as levels of a categorical fixed effect, and the mother receiving help, the mother giving help and year as random intercept effects.”

From R script: mDRorGRlatencyyears <- lmer(log(latency_day) ~ DRorGR + (1|year) + (1|CALFSMOTHER) + (1|DOEID), data = latency_grvsdr)

1. Since this is from the perspective of the mother that has received help and is subsequently offering help, I do not think it is necessary to include (1|DOEID) – i.e., the receiver, as a random factor.

2. It is important to add confounding variables in the model: Do mothers with male offspring allonurse as frequently as mothers with a female offspring (offspring sex)? Do older mothers allonurse as frequently as young mothers (mother's age)? Does the presence of closely related individuals influence the latency period (presence of kin – yes/no)? etc.

20. "For all models, there was no multicollinearity."

Please elaborate on which function was used to test for multicollinearity and provide the relevant stats and references.

It is difficult to comment on the results, as there are serious issues with data collection and statistical analyses.

21. "The interaction between the number of help received and pairwise genetic relatedness did not significantly affect the number of help given ($\beta \pm SE$: 0.25 ± 0.21, P = 0.24), which did not support our prediction of the interaction between the direct reciprocity and kin discrimination decision rules: the interaction was removed from the model."

As explained before, this is not acceptable, and potentially amounts to *P*-hacking.

22. "The number of help given increased as the number of help received increased ($\beta \pm SE$: 0.05 ± 0.02, P = 0.009, Fig 1A), which supported our prediction that reindeer mothers allonursed according to the direct reciprocity decision rule."

Looking at the graph, it seems that the model is underfitting the data. I would suggest the researchers to focus on and report effect sizes and not only *P* values. What was the R^2 for this and all the models? Potentially, the estimated coefficient could also be a measure of the effect size. Going by this, what does a 0.05 increase in help received mean? How biologically significant is it?

Please include units in the graphs and elsewhere. For e.g., in Figure 1 A, given and received allonursing bouts per and in Figure 1B, given allonursing bouts per?

23. "The number of help given was not significantly affected by the pairwise genetic relatedness ($\beta \pm SE$: 0.48 ± 0.60, P = 0.42, 290 Fig 1B), which did not support our prediction that reindeer mothers allonursed according to the kin discrimination decision rule."

Here again, report effect size, and it seems that the model is underfitting the data based on the graph. Additionally, why is the predictor range so small (pairwise genetic relatedness: -0.1 to 0.2)? How can a general inference about genetic relatedness be made from this limited range? That "reindeer mothers do not allonurse according to kin discrimination decision rule" is an inferential overreach.

24. "The interaction between RAFI and pairwise genetic relatedness did not significantly affect the likelihood to allonurse ($\beta \pm SE$: -0.43 ± 2.11, P = 0.84), which did not support our prediction of the interaction between the direct reciprocity and kin discrimination decision

rules: the interaction was removed from the model. The likelihood of allonursing increased as RAFI increased ($\beta \pm SE$: 0.57 ± 0.14, $P < 0.001$, Fig 2A), which supported our prediction that reindeer mothers allonursed according to the direct reciprocity decision rule."

As explained before, this analysis needs to be modified. Figure 2A is not meaningful. Always show graphs with the data and the fitted model.

25. "The likelihood of allonursing was not significantly affected by the pairwise genetic relatedness ($\beta \pm SE$: 0.38 ± 0.78, $P = 0.62$, Fig 2B), which did not support our prediction that reindeer mothers allonursed according to the kin discrimination decision rule."

This is inferential overreach. We only know (if we assume the model to be appropriate) that within the range of -0.1 to 0.2 pairwise genetic relatedness, relatedness does not have a significant effect on likelihood of allonursing.

26. "The overall number of help given increased as the overall number of help received increased ($\beta \pm SE$: 0.011 ± 0.004, $P = 0.006$, Fig 3), which supported our prediction that reindeer mothers allonursed according to the generalized reciprocity decision rule. The latency to give help after receiving help was shorter for generalized reciprocity than direct reciprocity by 18.30 days (mean ± SE latency for direct reciprocity: 19.42 ± 0.45 days (range: 1.00 – 64.09 days); mean ± SE latency for generalized reciprocity: 1.11 ± 0.04 days (range: 0.000046 – 16.31 days); model $\beta \pm SE$: -3.67 ± 0.07, $P < 0.001$, Fig 4)."

These are interesting results, but one can only comment on their soundness after the analysis is properly redone. Please include units in the graphs to help interpret the coefficients.

The authors have to take much care in writing the discussion section. The sentences in the introduction cannot be reproduced as such in the discussion. For e.g.,

From Discussion: The direct reciprocity decision rule explains reciprocal cooperation across several taxa, e.g. mammals (11,21–28) (e.g. vampire bats (22,23) and Norway rats (24–27)), birds (3,4), fishes (19,20), and microorganisms (30,31).

From Introduction: Direct reciprocity was reported across several taxa, including mammals (11,21–28) (e.g. vampire bats, *Desmodus rotundus* (22,23) and Norway rats, *Rattus norvegicus* (24–27,29)), birds (3,4), fishes (19,20), and microorganisms (30,31).

Please explain what has been found in the current study and how it relates to the existing knowledge regarding allonursing in reindeers in both wild and semi-domesticated populations. Please discuss if and how domestication influences the nature of allonursing. Discuss how the species' social organization and structure can influence allonursing (expectations for each hypothesis will have to be written out clearly) in the introduction, and whether the findings are in line with the expectation should be discussed. Importantly, please discuss all the limitations of the study and how they can be addressed in the future. Please discuss if and how the present study furthers what we know about allonursing, and more generally, cooperation in reindeer societies. Please also discuss if and how the current study contributes to our general knowledge about allonursing. Please do not discuss support for or against hypotheses for which data were not specifically collected and/or that were not formally tested (for e.g., the milk evacuation hypothesis). There are previous works on generalized reciprocity, therefore, I recommend the authors to cite those works (for e.g., Bohn *et al*. 2009: Pup guarding by greater spear-nosed

bats) and discuss them. After rework with a larger dataset if there is still support for generalized reciprocity, please discuss why other studies do not find support for it and what factors could have contributed to the lack of evidence and what factors could have led to generalized reciprocity in the study population.
