# Supplementary material for: Evidence suggesting that reindeer mothers allonurse according to the direct reciprocity and generalized reciprocity decision rules
Source: PLoS One. 2023 Dec 14;18(12):e0295497. doi: 10.1371/journal.pone.0295497 (PMC10721201; doi:10.1371/journal.pone.0295497)
Supplement: Attachment — Submitted filename: Review report.docx [file pone.0295497.s011.docx]

Article title: Evidence suggesting that reindeer mothers allonurse according to the direct reciprocity and generalized reciprocity decision rulesReview report.

This article represents the existence and nature of allosuckling behaviour in reindeer which could add important perspective to the allocare behaviour in mammals. The authors have used various statistical tests and models to justify their findings. Here are some minor issues which should be considered by the authors prior to the final publication of this manuscript.

Abstract:

Line 20-28: Reorder the statements according to your previous order of decision rules (line 18-20).

Material and Methods:

Line 169: One should be written in words. Same applicable for all single digit numbers.

Line 168: Although the author has mentioned that the detail method has been published previously, still some brief is necessary for the convenience of the reader.

Line 171: In 2012, the study animals were studied for 10 weeks, but for other years this information is missing.

Line 182: Unnecessary symbol.

Line: 288: Is this result supporting ‘direct decision rule’ or ‘generalized’? Please justify.
