# Supplementary material for: Evidence suggesting that reindeer mothers allonurse according to the direct reciprocity and generalized reciprocity decision rules
Source: PLoS One. 2023 Dec 14;18(12):e0295497. doi: 10.1371/journal.pone.0295497 (PMC10721201; doi:10.1371/journal.pone.0295497)
Supplement: Attachment — Submitted filename: Response to Reviewers.docx [file pone.0295497.s013.docx]

PONE-D-22-28248

Evidence suggesting that reindeer mothers allonurse according to the direct reciprocity and generalized reciprocity decision rules

PLOS ONE

Dear Dr. Engelhardt,

Thank you for submitting your manuscript to PLOS ONE. After careful consideration, we feel that it has merit but does not fully meet PLOS ONE’s publication criteria as it currently stands. Therefore, we invite you to submit a revised version of the manuscript that addresses the points raised during the review process.

Please submit your revised manuscript by Mar 18 2023 11:59PM. If you will need more time than this to complete your revisions, please reply to this message or contact the journal office at [plosone@plos.org](mailto:plosone@plos.org). When you're ready to submit your revision, log on to https://www.editorialmanager.com/pone/ and select the 'Submissions Needing Revision' folder to locate your manuscript file.

Please include the following items when submitting your revised manuscript:

- A rebuttal letter that responds to each point raised by the academic editor and reviewer(s). You should upload this letter as a separate file labeled 'Response to Reviewers'.
- A marked-up copy of your manuscript that highlights changes made to the original version. You should upload this as a separate file labeled 'Revised Manuscript with Track Changes'.
- An unmarked version of your revised paper without tracked changes. You should upload this as a separate file labeled 'Manuscript'.

If you would like to make changes to your financial disclosure, please include your updated statement in your cover letter. Guidelines for resubmitting your figure files are available below the reviewer comments at the end of this letter.

If applicable, we recommend that you deposit your laboratory protocols in protocols.io to enhance the reproducibility of your results. Protocols.io assigns your protocol its own identifier (DOI) so that it can be cited independently in the future. For instructions see: <https://journals.plos.org/plosone/s/submission-guidelines#loc-laboratory-protocols>. Additionally, PLOS ONE offers an option for publishing peer-reviewed Lab Protocol articles, which describe protocols hosted on protocols.io. Read more information on sharing protocols at <https://plos.org/protocols?utm_medium=editorial-email&utm_source=authorletters&utm_campaign=protocols>.

We look forward to receiving your revised manuscript.

Kind regards,

Anindita Bhadra, PhD
Academic Editor
PLOS ONE

Journal requirements:

When submitting your revision, we need you to address these additional requirements.

**AE_1**. Please ensure that your manuscript meets PLOS ONE's style requirements, including those for file naming. The PLOS ONE style templates can be found at

https://journals.plos.org/plosone/s/file?id=wjVg/PLOSOne_formatting_sample_main_body.pdf

and <https://journals.plos.org/plosone/s/file?id=ba62/PLOSOne_formatting_sample_title_authors_affiliations.pdf>.

**RE_AE_1: We revised our manuscript according to the style templates.**

**AE_2**. Thank you for including your ethics statement: " We designed this study in accordance with the Animal Ethics and Care Certificate of Concordia University (AREC-2010-WELA) and the Finnish National Advisory Board on Research Ethics.". To comply with PLOS ONE submissions requirements, please provide the following information in the Methods section of the manuscript and in the “Ethics Statement” field of the submission form (via “Edit Submission”): * Please indicate whether an animal research ethics committee prospectively approved this research or granted a formal waiver of ethics approval.* Please enter the name of your Institutional Animal Care and Use Committee (IACUC) or other relevant ethics board. Also include an approval number if one was obtained. * If anesthesia, euthanasia, or any kind of animal sacrifice is part of the study, please include briefly in your statement which substances and/or methods were applied. For additional information about PLOS ONE submissions requirements for ethics oversight of animal work, please refer to http://journals.plos.org/plosone/s/submission-guidelines#loc-animal-research Once you have amended this/these statement(s) in the Methods section of the manuscript, please add the same text to the “Ethics Statement” field of the submission form (via “Edit Submission”).

**RE_AC_2: We wrote the following in the methods section on lines 182-184 and in the Ethics Statement field of the submission form: “This study was carried out in strict accordance with the Animal Ethics and Care Certificate of Concordia University.  The protocol was approved by the Animal Ethics and Care Certificate of Concordia University (Protocol number: AREC-2010-WELA).” The Animal Ethics and Care Certificate of Concordia University evaluated the protocol to be carried out in Finland.**

**AE_3**. Thank you for stating the following financial disclosure:

“This project was supported by a doctoral bursary from the Fonds de recherche du Québec, Nature et technologies, a Northern Scientific Training Program award, and a QCBS Excellence Award granted to SCE. This project was also funded by RB's Natural Sciences and Engineering Research Council of Canada (https://www.nserc-crsng.gc.ca/index_eng.asp) Discovery Grant number 327505. The contributions of KR and ØH were funded by Reindeer Husbandry in a Globalizing North (ReiGN), which is a Nordforsk-funded (https://www.nordforsk.org/nordic-centre-excellence) “Nordic Centre of Excellence” (project number 76915).”

Please state what role the funders took in the study.  If the funders had no role, please state: ""The funders had no role in study design, data collection and analysis, decision to publish, or preparation of the manuscript.""

If this statement is not correct you must amend it as needed.

Please include this amended Role of Funder statement in your cover letter; we will change the online submission form on your behalf.

**Re_AE_3: We added the following in the cover letter: “****The funders had no role in study design, data collection and analysis, decision to publish, or preparation of the manuscript.”**

**AE_4**. We noted in your submission details that a portion of your manuscript may have been presented or published elsewhere. [No. The data have not been published elsewhere, however we combined data from 2 years of data collection. We published articles using parts of the data used in this manuscript. Some of data from Engelhardt et al. (2015) in Ethology is included in the data of the current manuscript. We describe in the methods that we revise the partial evidence suggesting that reindeer mothers allonurse according to the direct reciprocity decision rule (Engelhardt et al. 2015). The current manuscript now shows evidence suggesting that reindeer mothers do allonurse according to the direct reciprocity and generalized reciprocity decision rule. No results or figures were previously published or in pending manuscripts.

Please clarify whether this [conference proceeding or publication] was peer-reviewed and formally published. If this work was previously peer-reviewed and published, in the cover letter please provide the reason that this work does not constitute dual publication and should be included in the current manuscript.”

**RE_AE_4: None of the data were previously used to assess the generalized reciprocity decision rule nor the latency to give help after receiving help according to the direct reciprocity decision rule and the generalized reciprocity decision rule. The data from the 2013 field season were never before used to assess the direct reciprocity decision rule nor to assess the interaction between relatedness and the direct reciprocity decision rules. A subset of the 2012 data (i.e. the observations with calculated durations) was peer-reviewed and formally published in Engelhardt et al. (2015) to assess the reciprocal allonursing in Ethology. We added all of the data collected during 2012 to further assess if reindeer mothers allonurse according to the direct reciprocity and the kin discrimination decision rules. and generalized reciprocity decision rules. This manuscript is not a dual publication of Engelhardt et al. (2015), since i) we assess if reindeer mothers allonurse according to the direct reciprocity and generalized reciprocity decision rules, and ii) we add data from 2012 and 2013. A positive association between help received and help given within pairs of allonursing mothers, which supports the direct reciprocity decision rule, is completely novel for allonursing and more generally in alloparental care and communal breeding. Receiving help in general increased subsequent help given, since the overall number of help given increased as the overall number of help received, which suggests that reindeer mothers did not allonurse according to the generalized reciprocity decision rule: this result is completely novel for allonursing and more generally in alloparental and communal breeding. This manuscript and its results are novel in the field of study of allonursing and for the study of alloparental care, communal breeding social systems. With this manuscript, generalized reciprocity and direct reciprocity may also be considered for investigated in cooperative breeding social systems. No results or figures were previously published or in pending manuscripts.**

**AE_5**. We note that you have indicated that data from this study are available upon request. PLOS only allows data to be available upon request if there are legal or ethical restrictions on sharing data publicly. For more information on unacceptable data access restrictions, please see http://journals.plos.org/plosone/s/data-availability#loc-unacceptable-data-access-restrictions.

In your revised cover letter, please address the following prompts:

a) If there are ethical or legal restrictions on sharing a de-identified data set, please explain them in detail (e.g., data contain potentially sensitive information, data are owned by a third-party organization, etc.) and who has imposed them (e.g., an ethics committee). Please also provide contact information for a data access committee, ethics committee, or other institutional body to which data requests may be sent.

b) If there are no restrictions, please upload the minimal anonymized data set necessary to replicate your study findings as either Supporting Information files or to a stable, public repository and provide us with the relevant URLs, DOIs, or accession numbers. For a list of acceptable repositories, please see http://journals.plos.org/plosone/s/data-availability#loc-recommended-repositories.

We will update your Data Availability statement on your behalf to reflect the information you provide.

**RE_AC_5: We do not understand this comment.** **We made our data available in the initial submission, and the submission file read as follows “Yes - all data are fully available without restriction” and “All relevant data are within the manuscript and its Supporting Information files. We included 7 csv files and 1 .RData file. The .RData file can be opened in R or Rstudio with the function: load("latency_grvsdr.RData"). If you have a problem downloading the file, please contact the corresponding author. The Rscript is included and can be read in R or Rstudio.” The data files were also described in the Supplementing information section of the manuscript with some instructions to load the .RData file, and they were attached to the initial PDF submittion. This also affects the 3^rd^ comment to the Authors: “3. Have the authors made all data underlying the findings in their manuscript fully available?”. Both reviewers state that we provided the data, and Reviewer 2 also mentions the data their review.**

**The data is also available in the re-submission, and we changed the names of the files in accordance with the file names request of PLOS ONE. The data files are also described in the Supplementing information section of the revised manuscript. We added this information in the cover letter.**

6. Please include your full ethics statement in the ‘Methods’ section of your manuscript file. In your statement, please include the full name of the IRB or ethics committee who approved or waived your study, as well as whether or not you obtained informed written or verbal consent. If consent was waived for your study, please include this information in your statement as well.

**We wrote the following in the methods section on lines 182-184 and in the Ethics Statement field of the submission form: “This study was carried out in strict accordance with the Animal Ethics and Care Certificate of Concordia University.  The protocol was approved by the Animal Ethics and Care Certificate of Concordia University (Protocol number: AREC-2010-WELA).”**

Additional Editor Comments:

**AE_7**. This manuscript needs extensive revision. I am sorry that the decision took long, due to the difficulty of finding suitable reviewers. I agree with Reviewer 2 regarding the issue of data collection, and I suggest that the manuscript is revised keeping the detailed comments in mind. Analyzing the data for the two years separately is a good suggestion. I look forward to receiving the revised manuscript in the near future.

**RE_AE_7: We have revised the manuscript according to your comments and the reviewers’ comments. The analyses were also revised according to Reviewer 2’s comments.**

[Note: HTML markup is below. Please do not edit.]

Reviewers' comments:

Reviewer's Responses to Questions

**Comments to the Author**

1. Is the manuscript technically sound, and do the data support the conclusions?

The manuscript must describe a technically sound piece of scientific research with data that supports the conclusions. Experiments must have been conducted rigorously, with appropriate controls, replication, and sample sizes. The conclusions must be drawn appropriately based on the data presented.

Reviewer #1: Yes

Reviewer #2: No

2. Has the statistical analysis been performed appropriately and rigorously?

Reviewer #1: Yes

Reviewer #2: No

3. Have the authors made all data underlying the findings in their manuscript fully available?

The [PLOS Data policy](http://www.plosone.org/static/policies.action#sharing) requires authors to make all data underlying the findings described in their manuscript fully available without restriction, with rare exception (please refer to the Data Availability Statement in the manuscript PDF file). The data should be provided as part of the manuscript or its supporting information, or deposited to a public repository. For example, in addition to summary statistics, the data points behind means, medians and variance measures should be available. If there are restrictions on publicly sharing data—e.g. participant privacy or use of data from a third party—those must be specified.

Reviewer #1: Yes

Reviewer #2: No

4. Is the manuscript presented in an intelligible fashion and written in standard English?

PLOS ONE does not copyedit accepted manuscripts, so the language in submitted articles must be clear, correct, and unambiguous. Any typographical or grammatical errors should be corrected at revision, so please note any specific errors here.

Reviewer #1: Yes

Reviewer #2: Yes

5. Review Comments to the Author

Please use the space provided to explain your answers to the questions above. You may also include additional comments for the author, including concerns about dual publication, research ethics, or publication ethics. (Please upload your review as an attachment if it exceeds 20,000 characters)

Reviewer #1: This article represents the existence and nature of allosuckling behaviour in reindeer which could add important perspective to the allocare behaviour in mammals. The authors have used various statistical tests and models to justify their findings. Please see the review report for some minor issues which should be considered by the authors prior to the final publication of this manuscript.

Reviewer #2: The review comments have been included as an attachment, which includes an overview of the comments as well as detailed comments for all the sections of the manuscript. Please refer to the attached PDF for a complete review.

6. PLOS authors have the option to publish the peer review history of their article ([what does this mean?](https://journals.plos.org/plosone/s/editorial-and-peer-review-process#loc-peer-review-history)). If published, this will include your full peer review and any attached files.


If you choose “no”, your identity will remain anonymous but your review may still be made public.


**Do you want your identity to be public for this peer review?** For information about this choice, including consent withdrawal, please see our [Privacy Policy](https://www.plos.org/privacy-policy).

Reviewer #1: No

Reviewer #2: No

[NOTE: If reviewer comments were submitted as an attachment file, they will be attached to this email and accessible via the submission site. Please log into your account, locate the manuscript record, and check for the action link "View Attachments". If this link does not appear, there are no attachment files.]

While revising your submission, please upload your figure files to the Preflight Analysis and Conversion Engine (PACE) digital diagnostic tool, <https://pacev2.apexcovantage.com/>. PACE helps ensure that figures meet PLOS requirements. To use PACE, you must first register as a user. Registration is free. Then, login and navigate to the UPLOAD tab, where you will find detailed instructions on how to use the tool. If you encounter any issues or have any questions when using PACE, please email PLOS at [figures@plos.org](mailto:figures@plos.org). Please note that Supporting Information files do not need this step.

Reviewer 1

Article title: Evidence suggesting that reindeer mothers allonurse according to the direct reciprocity and generalized reciprocity decision rulesReview report.

This article represents the existence and nature of allosuckling behaviour in reindeer which could add important perspective to the allocare behaviour in mammals. The authors have used various statistical tests and models to justify their findings. Here are some minor issues which should be considered by the authors prior to the final publication of this manuscript.

Abstract:

**R1_1:** Line 20-28: Reorder the statements according to your previous order of decision rules (line 18-20).

**RE_R1_1: We reordered the decision rules on lines 25-27 as follows: “****Cooperation can be explained by three evolved decision rules: 1) direct reciprocity, i.e. help someone who previously helped you, 2) kin discrimination, i.e. preferentially direct help to kin than to non-kin, and 3) generalized reciprocity, i.e. help anyone if helped by someone.” and on lines 98-104: “Cooperation can be explained by three evolved, mechanistic decision rules: 1) direct reciprocity, i.e. help someone who previously helped you (if A helped B, B helps A) [20,21], 2) kin discrimination is the differential treatment of conspecifics as a function of their genetic relatedness to the actor [20,24], and at the proximate mechanism level it implies a decision rule: preferentially direct help to related kin than to non-kin (if A and B are kin, but A and C are non-kin, A helps B), and 3) generalized reciprocity, i.e. help anyone if helped by someone (if A helped B, B is more likely to help anyone) [20,21].”**

Material and Methods:

**R1_2:** Line 169: One should be written in words. Same applicable for all single digit numbers.

**RE_R1_2: We wrote all single digit numbers in words throughout the manuscript.**

**R1_3:** Line 168: Although the author has mentioned that the detail method has been published previously, still some brief is necessary for the convenience of the reader.

**RE_R1_3: We added more details about the methods.**

**We wrote the following on lines 186-191: “****The Kutuharju Field Reindeer Research Station is a research station, and the reindeer are owned by reindeer herders, who manage the size of groups and in which sections of the station different groups will roam during the calving season. The herd is also managed to avoid inbreeding. Yearly, the semi-domesticated female reindeer are herded into open fenced paddocks (approximately 10 ha), where data on the birth date, calf sex, and mother-calf assignments were obtained. This population that has been monitored since 1969** **[96].”**

**We wrote the following on lines 204-211: “The manager of the herd maintained a population of females ranging between 2-13 yr in 2012 (mean ± SD = 7.32 ± 3.21 yr) and between 2-12 yr in 2013 (mean ± SD = 5.93 ± 3.76 yr). The age of mothers ranged from 2-13 yr in the 2012 group (mean ± SD = 8.25 ± 2.96 yr). The age of mothers ranged from 3-8 yr in the 2013 closely-related group study group (mean ± SD = 6.00 ± 2.51 yrs), and th age of mothers ranged from 5-11 yr in the 2013 distantly-related group (mean ± SD = 8.12 ± 2.42 yr). Mother-offspring pairs were assigned within 24-48 hours of parturition. Researchers fixed collar tags of different colours, with numbers inscribed, to individuals for identification.”**

**We wrote the following on lines 219-220: “****Previous studies of allosuckling in reindeer reported that most mothers allonursed and most offspring allosuckled [98,102].”**

**We wrote the following on lines 232-257: “****All mothers were habituated to human presence. Observations were conducted inside the paddock at a distance ranging from 5 to 50 meters from animals by 3 trained observers in 2012 and 1 trained observer in 2013. Binoculars were used to reliably record observations of solicitations, agonistic interactions, and identify individuals. Observations of nursing and allonursing solicitations were collected on data collection sheets using** **behaviour sampling with continuous recording [103]. For each solicitation, the occurrence of nursing and allonursing and the identity of the mother and offspring were recorded. A solicitation was scored as an attempt when an offspring brought its muzzle within a head from a mother’s udder, and the mother did not allow the offspring to be nursed (e.g. walking away, kicking, head threat, chasing). A solicitation was scored as a rejection when the offspring suckled for less than 5 seconds [100], and the termination was due to the mother not allowing the offspring to be nursed any longer. A solicitation was scored as successful (i.e. a bout) when an offspring suckled for 5 seconds or more and ended when the offspring no longer grasped the mother’s udder. We selected a 5 seconds cut-off based on previous nursing and allonursing research with reindeer [98,104], and a 5 seconds cut-off has also been selected to study nursing and allonursing in red deer, *Cervus elaphus* [16,105], fallow deer, *Dama dama* [106], cows, *Bos taurus* [100], bactrian camels (*Camelus bactrianus*) [101], zebras, *Equus greyvi* [107], bighorn sheep, *Ovis canadensis* [108].**

**Four behaviour sampling sessions of agonistic interactions of one hour each, using continuous recording, were conducted daily in 2012 [103]. Throughout each observation day in both 2012 and 2013, agonistic interactions were opportunistically scored using ad libitum sampling and continuous recording methods. An agonistic interaction was recorded as resolved when an individual showed a submissive behaviour (“lose”), and the other did not (“win”). Unresolved agonistic interactions were recorded as unresolved when neither animal showed a submissive behaviour. The agonistic interactions scored were displacement, head threat, push, chase, kick, boxing, and other interactions [109,110], and their associated submissive behaviours were scored as ‘flee’ or ‘walk away’ if submission occurred.** **The rank of female reindeer is fairly stable throughout the year [111], except for a very short time immediately following the shedding of antlers [110–112].”**

**R1_4:** Line 171: In 2012, the study animals were studied for 10 weeks, but for other years this information is missing.

**RE_R1_4: We wrote the following on line 194-200: “In 2012, the first 25 offspring born and their mothers were selected and studied for 65 observations days over 10 weeks [87–89].** **In 2012, the 25 offspring were born between May 4^th^ and May 13^th^.** **In 2013, we studied two groups for 25 observations over five weeks and each group consisted of eight mother-offspring pairs based on their pairwise genetic relatedness, forming two groups at the extremes of pairwise genetic relatedness: one group of closely, genetically related mothers and one group of distantly, genetically related mothers** **[79].”**

**R1_5:** Line 182: Unnecessary symbol.

**RE_R1_5: This is an issue when the word document was converted to a PDF file during the submission process. Our word document does not have that symbol in “cut-off”. The symbol should be an hyphen between cut and off.**

**R1_6:** Line: 288: Is this result supporting ‘direct decision rule’ or ‘generalized’? Please justify.

**RE_R1_6: This result supports the direct reciprocity decision rule as follows on lines 467-471: “The number of help given increased as the number of help received increased (main effect: *β ± SE*: 0.05 ± 0.02, *P* = 0.0096, IRR (95% CI): 1.05 (1.01 – 1.09), Fig 1A) in the 2012 group. For a one unit increase in the number of help received, the number help given is expected to increase by a factor of 1.05 (95% CI for IRR: 1.01 – 1.09). If a mother received help one, 10 or 15 times, the mother is expected to give help 2.61, 4.10 and 5.26 times, respectively.” This is at the dyadic level of analysis between pairs of reindeer mothers. For generalized reciprocity, there is no dyadic level of analysis between pairs of mothers.**

Reviewer 2

Overview of comments:

The authors have attempted to study three hypotheses to understand allonursing in semi-domesticated reindeer: direct reciprocity, generalized reciprocity, and kin selection. The authors have also attempted to compare direct and generalized reciprocity in the study population, which seemingly present interesting results, as well as the interaction between direct reciprocity and kin selection.

The authors have cited relevant literature for the hypotheses but have not discussed them adequately. Apart from discussing various hypotheses for allonursing, it will be useful to also briefly discuss the various factors – such as mother’s age, offspring sex, offspring age, etc., that are known to affect allonursing in wild and/or domesticated reindeer populations. It is necessary to describe the study species’ social organization and structure and how it differs from the study population, as allonursing should be understood in the context of the species’ biology.

Unfortunately, the study suffers from severe methodological problems. The data on allonursing bouts were collected over a span of 10 and 5 weeks in 2012 and 2013 following two different methods. Overall, I do not think it is appropriate to pool 2012 and 2013 data during analysis. Nor should that data on two groups be pooled for 2013; each group should be a data point. Though it is theoretically possible to use a nested model (as each individual appears in only one year), the method of data collection is very different between 2012 and 2013, so it will not be appropriate to pool data across years or groups. This, I believe, is one of the major problems with the study.

The best that can be done is to analyse 2012 data separately and 2013 data separately, however, this will require different methods of statistical analysis. I would even recommend the authors to use only the 2012 data and analyse them only for direct and generalized reciprocity, as the predictor range is very narrow for genetic relatedness. However, the 2012 and 2013 data are already published.

The methods, primarily, and the results section will have to be written in much more detail to make it possible for anyone to understand and replicate the study. The conclusions are drawn from inappropriate data analysis and through extrapolating the results, lead to the conclusions not being supported by the data. Specific recommendations are written below (in blue).

The manuscript in its current form is not suitable for publication due to major problems with data collection protocol including artificially constructing groups that can easily lead to erroneous results, insufficient data to test functional hypotheses, and improper analyses on previously published data. Unfortunately, the manuscript will have to be rejected.

**Thank you for comments. We accounted for your comments in the revised manuscript. We expanded the description of the methods. We revised the analyses.**

Detailed comments:

**R2_1:** 1. “Altruism, i.e. a behaviour costly to the actor and beneficial to the recipient (1), was one of Darwin’s greatest challenges to his theory of natural selection.”

Since there is a vast body of work on altruism subsequently, starting the manuscript with an allusion to Darwin is like setting up a straw-man.

**RE_R2_1: We revised the sentence on lines 78-79: “Altruism, i.e. a behaviour costly to the actor and beneficial to the recipient** **[19].”**

**R2_2:** 2. “Reciprocity is an apparently helpful behaviour,...”

Why is it apparently helpful as opposed to helpful?

**RE_R2_2: We revised our definition of reciprocity on lines 91-93: “Reciprocity is an apparently cooperative trait or behaviour, which benefits the recipient of the help at a cost to the actor, that increases the probability of the actor of the helpful behaviour receiving help in return from the same or different social partners [20,21].” We based our definition on Taborsky, Frommen and Riehl (2016) and Taborsky, Cant and Komdeur (2021). Taborsky et al. (2021) provide the following definition of reciprocity “This is essentially a proximate (i.e. mechanistic) concept implying certain cost/benefit relationships. At the ultimate (i.e. evolutionary) level, this term refers to an apparently cooperative trait or behaviour that benefits a receiver of the act at immediate costs to the actor. At the same time, it increases the probability of receiving benefits in return, from the same or different partners. Reciprocation is hence intrinsically altruistic and prone to cheating. At the proximate level, there are three forms of reciprocity implying different decision rules: …”. Taborsky et al. (2016) explain it as follows: “Social semantics is a contentious issue. Much controversy about the importance of different evolutionary mechanisms of cooperation results just from divergent understanding of terms. Therefore, to minimize the danger of creating misunderstandings, we use everyday language terms in the way most closely resembling the connotation they have in daily life.”. In the book by Taborsky et al. (2021), they provide a more detailed explanation for using “everyday language terms in the way most closely resembling the connotation they have in daily life”**

**R2_3:** 3. “Cooperation was better explained by direct reciprocity than relatedness (22,24,48) and generalized reciprocity (38) in empirical studies.”

There are a lot of studies of cooperation with evidence for both relatedness and reciprocity. The introduction should be shortened and follow a logical flow. Also, please remove details of various hypotheses that are not being tested in the paper. They can just be listed.

**RE_R2_3: We agree that there are many studies assessing the impact of relatedness on cooperation. We added sentences to introduce empirical studies and comparative analyses of the association between relatedness and cooperation on lines 119-124: “For empirical studies in taxa with a high social complexity, i.e. eusocial and cooperative breeding social systems, the association between relatedness and cooperation was positive [63–66], negative [67–69]** **and neutral [70–76]. In a comparative analysis of cooperatively breeding vertebrates, helping rates increased positively with relatedness, which explained 10% of the variation in helping rates [77], whereas altruism declined with increasing intragroup relatedness in a** **comparative analysis of eusocial hymenoptera [78].”**

**We agree that there are many studies of cooperation with evidence of reciprocity, and we have the following sentences in the introduction on lines 109-116: “Reciprocal cooperation has been reported in vertebrates and invertebrates [20,21,37,38]. Direct reciprocity was reported across several taxa, including mammals [28,39–46] (e.g. vampire bats, *Desmodus rotundus* [40,41] and Norway rats, *Rattus norvegicus* [42–45,47]), birds [20,21], fishes [37,38], and microorganisms [48,49]. Generalized reciprocity was reported in humans [50,51], female Norway rats [44,52], dogs, *Canis familiaris* [53], capuchin monkeys, *Sapajus apella* [54], however generalized reciprocity was not supported in male Norway rats [55], longtailed macaques, *Macaca fascicularis* [56] and in vampire bats [40].”. We also have the following sentences in the introduction on lines 126-128: “Cooperation can be explained by reciprocity and relatedness, and their fitness pay-offs are correlated [21]. Theoretical models predict i)** **a difference [81], and ii) an interaction in the contribution of direct reciprocity and relatedness for the evolution of cooperation [82,83].”**

**We very briefly listed the allonursing hypotheses and significantly reduced length of the allonursing hypotheses on lines 69-77: “The misdirected parental care, i.e. either due to milk-theft and mismothering [7], kin selection [7], reciprocity [7], compensation [16], improved nutrition [6,17], milk evacuation [7], neuroendocrine [18] and immunological function [13] hypotheses were proposed to explain why females allonurse the offspring of other mothers. The kin selection hypothesis proposes that lactating females preferentially allonurse closely over distantly related offspring, providing inclusive fitness benefits [7]. The reciprocity hypothesis proposes that two females achieve a higher fitness when nursing each other’s offspring to a similar extent than when they do not share milk [7]. Reciprocal allonursing is the reciprocated feeding of offspring.”**

**We reorganized the introduction. The introduction now begins with an introduction of cooperative breeding, alloparental care, allonursing and allonursing hypotheses. The second paragraph introduces altruism, help, cooperation, kin-selection and reciprocity. The third paragraph introduces cooperation as an emergent property of evolved decision rules and introduces the decision rules of cooperation. The fourth paragraph introduces the past results for allonursing in reindeer to place readers in the context of the study, and our fifth paragraph introduces the research questions and predictions with regards to the decision rules of cooperation. We think each paragraph introduces an important component for readers, so we can introduce the research question and predictions in the fifth paragraph.**

**R2_4:** 4. “Relatedness did not influence the odds of allonursing (73), however a group of closely-related mothers allonursed each other’s offspring more often than a group of distantly-related mothers at the extremes of pairwise genetic relatedness (72).”

Given this, why is genetic discrimination being tested again in this paper?

**RE_R2_4: We wrote the following on lines 85-96: “****The indirect fitness benefits as a result of helping appear to be smaller than first expected or may be underestimated, and the direct fitness benefits as a result of help appear to be greater than first expected and may even be greater than the indirect fitness benefits** **[21,25,26]. Both direct and indirect fitness can interact [21]. Help between kin is well explained by** **indirect fitness [27], which cannot explain help between non-kin (e.g. reciprocity) [20,21,25]. Cooperation between unrelated individuals is common across a wide range of taxa [20,21,28].** **Reciprocity is an apparently cooperative trait or behaviour, which benefits the recipient of the help at a cost to the actor, that increases the probability of the actor of the helpful behaviour receiving help in return from the same or different social partners [20,21]. For reciprocity, the helping costs for the actor should be low; benefits for the receiver should be high; and the probability of receiving help in return for help given should be high [28]. Reciprocal interactions can also occur between related and unrelated individuals, such that the correlated pay-offs can interact with relatedness [21].”**

**We wrote the following on lines 117-133: “****Most of the evidence for the importance of kin selection for the evolution of cooperation is correlational****. For empirical studies in taxa with a high social complexity, i.e. eusocial and cooperative breeding social systems, the association between relatedness and cooperation was positive [63–66], negative [67–69] and neutral [70–76]. In a comparative analysis of cooperatively breeding vertebrates, helping rates increased positively with relatedness, which explained 10% of the variation in helping rates [77], whereas altruism declined with increasing intragroup relatedness in a comparative analysis of eusocial hymenoptera [78].** **Several studies that experimentally manipulated relatedness found that relatedness reduced cooperation rather than increase it [42,69,79,80], which differs with kin selection theory. Cooperation can be explained by reciprocity and relatedness, and their fitness pay-offs are correlated [21]. Theoretical models predict i) a difference [81], and ii) an interaction in the contribution of direct reciprocity and relatedness for the evolution of cooperation [82,83]. Cooperation was better explained by direct reciprocity than relatedness [40,42,84] and generalized reciprocity [56] in empirical studies. Direct reciprocity and generalized reciprocity can co-exist, such as in humans [50] and female Norway rats [44,52]. Theoretically, cooperation usually stems from the interaction of multiple evolutionary mechanisms [81–83,85,86]. Very little is known about the relative importance of these 3 decision rules****, except in male Norway rats [42,55].”**

**We wrote the following on lines 153-161: “****There is an on-going debate over the utility of kin selection and alternative mechanisms [20,21,34,91–95], which reveals the need to experimentally test combinations of evolved decision rules to further understand the interactions and relative importance of the mechanisms responsible for the evolution of cooperation. To improve on the superficial appreciation of the involved mechanisms responsible for the evolution of cooperation, i) we address the evolved, mechanistic decision rules of direct reciprocity, generalized reciprocity and kin discrimination responsible for cooperation, and ii) we combine evolved decision rules of cooperation to assess the interaction between the direct reciprocity and kin discrimination decision rules.”**

**R2_5:** 5. “Although we previously found partial support for reciprocal allonursing in reindeer (70), we need to assess i) the evolved, mechanistic decision rules of direct reciprocity, generalized reciprocity and kin discrimination responsible for cooperation, and ii) the interaction between the direct reciprocity and kin discrimination decision rules.”

Why this needs to be assessed should be the crux of the introduction.

**RE_R2_5: See our reply RE_R2_4.**

**R2_6:** 6. “In 2012, the first 25 offspring born and their mothers were selected and studied for 10 weeks.”

What was the reason for choosing only the first 25 offspring?

**RE_R2_6:** **The Kutuharju Field Reindeer Research Station is a research station, and the reindeer are owned by reindeer herders, who manage the size of groups and in which sections of the station different groups will roam during the calving season. The first 25 mothers and their offspring represented a number of mother-offspring pairs that the reindeer herders agreed to for 10 weeks: it was a negotiation between the researchers (authors) and the reindeer herders. Yearly, the semi-domesticated female reindeer are herded into open fenced paddocks prior to parturition and kept there for 3–5 weeks. The study group was separated from the rest of the herd for 10 weeks.**

**We wrote the following on lines 186-191: “The Kutuharju Field Reindeer Research Station is a research station, and the reindeer are owned by reindeer herders, who manage the size of groups and in which sections of the station different groups will roam during the calving season. The herd is also managed to avoid inbreeding. Yearly, the semi-domesticated female reindeer are herded into open fenced paddocks (approximately 10 ha), where data on the birth date, calf sex, and mother-calf assignments were obtained. This population that has been monitored since 1969 [96].”**

**R2_7:** 7. “In 2013, we obtained 2 groups and each group consisted of 8 mother-offspring pairs based on their pairwise genetic relatedness, forming 2 groups for 5 weeks at the extremes of pairwise genetic relatedness: one group of closely, genetically related mothers and one group of distantly, genetically related mothers.”

From reading the methods section of this manuscript and those of the cited works from the research group (Engelhardt *et al*. 2014, 2015, 2016 a,b or references 70-73), I understand that the methods followed in 2012 and 2013 are different.

In 2012, 25 mother-offspring pairs were separated from the larger herd, and behavioural observations were taken on them. In 2013, a group each of closely and distantly related mothers (along with their offspring) were formed, and behavioural observations were taken separately on these groups.

What was the necessity to form two separate groups in 2013? If it is to check for the effect of relatedness, forming two separate groups is not necessary. Relatedness can be treated as a continuous variable and its effect on the number of help given or received can be analysed.

Ultimately, the researchers have pooled all the data from 2012 and 2013 to analyse the effect of direct and generalized reciprocity and kin discrimination. This again brings up the question: why form two groups at all in 2013?

Please provide the average ± 95%CI as well as range of pairwise genetic relatedness for both the years as well as groups.

Year was included as a random factor in only two of the four models run. The effect of year was not included in the results. I suggest the authors to include the effect of all the fixed and random factors included in all the models in the results section.

The period of study between the two years is also different (2012: 10 weeks, 2013: 5 weeks). This would mean that calves born in 2012 would have been followed for a longer duration of their infancy than the calves born in 2013. If I understand correctly, the ages of offspring of the two groups of 2013 were similar but not the ages of offspring of 2012 and 2013. Please provide justifications for how the difference in the length of infancy period covered would not affect the results. As far as I understand, age of offspring was not included as a factor in any of the models.

**RE_R2_7: We expanded the methods for both the 2012 and 2013 studies. Please see our response RE_R1_3. The period of study and the design of the studies differ between both years.**

**Most studies of the importance of relatedness are correlational, and few studies manipulated relatedness (see our response RE_R2_4). We manipulated relatedness in 2013 by choosing reindeer mothers to form 2 groups at the extremes of pairwise genetic relatedness in the population. Pairwise genetic relatedness as a continuous variable did not significant effect the likelihood of a successful allosuckling bout, and we wanted to assess if relatedness at the extremes of relatedness in the population affected help allonursing given. We wrote the following on lines 200-202: “We manipulated relatedness in 2013 by choosing reindeer mothers to form 2 groups at the extremes of pairwise genetic relatedness in the population to assess if relatedness at the extremes of relatedness in the population affected help allonursing given [79].”**

**We wrote the following for pairwise genetic relatedness on lines 269-272: “The LRM estimates of pairwise genetic relatedness were calculated for the 3 groups (2012: mean ± SE = -0.009 ± 0.003 , 95% CI = -0.016 – 0.002, range = -0.144-0.239; 2013 closely-related: mean ± SE = 0.024 ± 0.011 , 95% CI = 0.00059 – 0.047, range = -0.072-0.152; 2013 distantly-related: mean ± SE = -0.023 ± 0.007 , 95% CI = -0.038 – -0.009, range = -0.071-0.062).”**

**We compared the age of offspring in the 3 groups. To compare the age of offspring, we chose to compare their age on May 30^th^ of 2012 or 2013. In 2013, the study started on May 30^th^, and we used this day to compare offspring age. We wrote the following on lines 306-310: “****To assess differences in the age of offspring between groups, we ran a generalized linear mixed model with a Poisson distribution with age of the offspring on May 30^th^ in either 2012 or 2013 as the response and group as the fixed effect. Year was a random intercept effect. Post hoc Tukey comparisons were performed for multiple comparisons (i.e. distantly-related vs closely-related groups), and we corrected the alpha to 0.025 to account for multiple testing.” We presented the mean, SE and 95% confidence intervals and the model results on lines 443-450: “The age of offspring on May 30^th^ in the 3 groups was calculated (2012: mean ± SE = 22.08 ± 0.47, 95% CI = 21.11 – 23.05; 2013 closely-related: mean ± SE = 15.63 ± 0.86 , 95% CI = 13.58 – 17.67; 2013 distantly-related: mean ± SE = 15.38 ± 1.84 , 95% CI = 11.02 – 19.73). The offspring in the closely-related group were younger on May 30^th^ 2013 than the offspring on May 30^th^ 2012 (*β ± SE*: -0.35 ± 0.10, *P* < 0.001), and the offspring in the distantly-related group were younger on May 30^th^ 2013 than the offspring on May 30^th^ 2012 (*β ± SE*: -0.36 ± 0.10, *P* < 0.001). There was no difference in the age of offspring in the closely- and distantly-related groups on May 30^th^ 2013 (Post-hoc Tukey distantly-related vs closely-related: Estimate *± SE*: -0.02 ± 0.13, *P* = 0.99).”**

**Offspring age difference is included in our response RE_R2_14.**

**We included all the model results in the main text, including the random effects and effect sizes (incidence rate ratio and odds ratio with 95% CI, and the conditional R^2^ of the model).**

**R2_8:** 8. “The study animals were separated from the herd until the end of the study, after which they joined the rest of the herd.”

What was the reason for separating the study animals from the larger herd? Would it not artificially restrict who was available for allonursing and bias the results?

Would it also not influence the natural behaviour of the semi-domesticated animals? I understand that comparing the behaviours of mothers before and after separation from the larger herd would not be possible due to the study design. However, were the behaviours of separated mothers compared with that of mothers who were not separated? Were the behaviours of mothers compared after separation and after rejoining? If the authors can provide justifications for separation not affecting individual behaviour, it would be helpful.

Separating the females from the herd for study will also introduce confounding factors. The factors influencing allonursing are likely to be highly dependent on evolved social systems. Therefore, the study should be situated within social groupings that the females are used to. Inferences made by separating females from the original social grouping may not hold at all in a more natural scenario. Much more detail should be provided about the social structure of reindeer.

Out of curiosity: Does pairwise familiarity, and not relatedness, affect allonursing in the study population? Is it difficult to tease apart the effect of familiarity vs relatedness?

**RE_R2_8: Reindeer herds in the wild are very large, and there is no way to study allonursing hypotheses in wild reindeer. There are multiple reasons why allonursing is not study in wild reindeer/caribou. Firstly, wild herds are so large that it is not possible to identify individuals and nursing and allonursing can only be observed on the outside perimeter of the herd. This would be an exercise of stating whether or not wild reindeer/caribou do allonurse and how many offspring were allonursed during a nursing bout. Secondly, wild reindeer/caribou must be observed from a long distance, otherwise they move away from the researcher. Domesticated reindeer and caribou do not exist. The Sámi herders have managed to semi-domesticate reindeer, and there is no evidence of semi-domestication in caribou. Reindeer and caribou are the same species: *Rangifer tarandus*.**

**The important question for the evolution of cooperation in reindeer is if reindeer can allonurse according to the direct reciprocity, generalized reciprocity and kin discrimination decision rules. These decision rules are evolved decision rules.**

**The reindeer at the research station are owned and managed by Sámi reindeer herders. We negotiated with them to run the studies in 2012 and 2013. Each calving season, the reindeer manager separates the reindeer into groups. Throughout the year, the reindeer are herded and weighed once per month. The reindeer manager separated the reindeer before allonursing began. Before the rut season, the reindeer manager separates the herd into groups. This study population is often used for research, and the herd is often manipulated. As such, the reindeer are habituated to be herded, weighed and separated into groups. We spoke with several Sámi reindeer herders about allonursing and our results, and we were told that the occurrence of allonursing we report was typical. This is a semi-domesticated population of reindeer. Given the manipulations of the herd, we did not think that individual behaviours would be affected by the formation of groups and separation of individuals.**

**We did not compare the behaviours of mothers after separation and after rejoining. When the study reindeer joined the rest of the herd, they were in a very large enclosure, approximately 15 km^2^. If the herd is too large, we can only see the allonursing bouts on the outside of the herd, not within the herd. During the study, we occasionally saw one group for 30 min, and we observed allonursing, however we did not record the behaviours since we were recording behaviours for the studies.**

**Researchers reported that reindeer offspirng suckled 3 times/hr (Espmark, 1971c) or 1.7 times/hr (Lavigueur & Barrette, 1992) during the first week, and the frequency of suckling decreased throughout the lactation period. Researchers have reported allosuckling and allonursing in reindeer (Espmark, 1971c; Lavigueur & Barrette, 1992; Marken, 2003. Espmark (1971c) recorded 85 allonursing bouts, where 14 of the 15 mothers allonursed and all offspring solicited allosuckling bouts. In another study, 6 of 6 mothers allonursed and each of their offspring were allonursed, and there were 290 allonursing bouts (Marken, 2003). The hypothesized causes of allosuckling and allonursing were not tested in these studies. No variables were tested in these studies, so there was no knowledge about the effect of variables on allonursing in wild and semi-domesticated reindeer populations.**

**All female reindeer were familiar with each other, so there is no effect of familiar on allonursing. There is no way to tease apart familiarity.**

**R2_9:** 9. “A successful allonursing bout was considered terminated when an offspring’s muzzle and a lactating female's udder were not in contact for 20 s or more.”

How was the 20 s cut-off decided? Please state the reasons.

Though successful and unsuccessful allonursing bouts are differentiated, how are independent and non-independent allonursing bouts differentiated? If independent data points are not selected for the analyses, one would run into autocorrelation (pseudoreplication) issue.

**RE_R2_9: First, we spoke with Sámi reindeer herders who told us that the duration between allonursing bouts were long, much longer than 20 s: this was Traditional Indigenous Knowledge. Second, Prof. Øystein Holand had observed reindeer nursing and some allonursing in an enclosure near his University office, and he had noticed the duration between allonursing bouts were long, i.e. much longer than 20 s. Thirdly, the first author of this manuscript noticed a few nursing and allonursing bouts before the rut season in 2010, and no new attempts occurred after 20 s of an offspring’s muzzle and a lactating female’s udder were no longer in contact, and there were no nursing and allonursing bouts between that lactating female and her offspring on that day after the lactating female ended the bout by walking away. Thus, we decided on a 20 s cut-off time, so we could observe other interactions.**

**For the models for direct reciprocity and kin discrimination decision rules in the revised submission, we ran separate models for each of the 3 groups and included 2 random intercept effects, i.e. the identity of both lactating females (id1 and id2), to account for pseudoreplication. The model for the comparison between the direct reciprocity and generalized reciprocity decision rules for the latency to give help after receiving help included the 2 random intercept effects (the identity of lactating females allonursing and the year) to account for pseudoreplication.**

**R2_10:** 10. “Reindeer nursing bouts last 2.7 times longer than allonursing bouts (nursing: 41 s; allonursing: 15 s)”

Please specify the average durations of nursing and allonursing bouts along with 95% CI and sample sizes.

Out of curiosity: If the duration of allonursing was analysed with respect to the degree of pairwise genetic relatedness between the mothers, would the results of this paper have been different? Is it possible for mothers to allonurse to similar frequencies regardless of their relatedness, but allonurse to differing durations with respect to their relatedness?

Regarding relatedness: Is there any evidence to suggest that reindeers identify their maternal and/or paternal kin either in the wild or in captivity? Even if there isn’t any evidence, I suggest that the authors include a couple of sentences about kin recognition in the introduction when introducing the kin discrimination hypothesis.

**RE_R2_10: We wrote the following on lines 438-442: “****In 2012, the mean duration of nursing bouts was 2.71 times longer than that for allonursing bouts (nursing bouts: 40.23 ± 0.63 s, 95% CI = 39.00 – 41.46 s, N = 3396 nursing bouts; allonursing bouts: 14.85 ± 0.29 s, 95% CI = 14.29 – 15.42 s, N = 1022 allonursing bouts). In 2013, the mean duration of allonursing bouts was 16.82 ± 0.74 s (95% CI = 15.37 – 18.27 s, N = 89 allonursing bouts).”**

**Although we recorded the frequency of allonursing bouts, we did not record the duration of all allonursing bouts. To run an analysis with the duration given and duration received, we removed dyads with unknown durations: this removed 59 dyads. Thus, we reported the frequency of help given and help received in the manuscript rather than rely on the duration help given and help received. We present the results for duration below, and they are not included in the revised manuscript.**

**We assessed if the duration of allonursing bouts given (i.e. the duration of help given), at the dyadic level, was influenced by the duration of allonursing bouts received (i.e. the duration of help received) and relatedness. We ran model separately by group i) 2012, ii) 2013 closely-related group, and iii) 2013 distantly-related group as the reviewer commented about. To summarize, relatedness did not significantly affect the duration of help given in any of the models that follow. We ran lmer models with the identities of both mothers as random effects to assess if the duration of allonursing bouts given (i.e. the duration of help given), at the dyadic level, was influenced by the duration of allonursing bouts received and relatedness. To meet the assumption that the residuals were normally distributed, we log-transformed the duration of help given.**

**2012 group. We first ran the model with an interaction between the duration of help received and relatedness, but the interaction was not significant and was removed from the model (see out response RE_R2_14 paragraph 6 for an explanation of this approach). The duration help given was the response variable, and we the duration of help received, relatedness, absolute rank difference, similarity of offspring sex (1 = same sex, 0 = different sex), difference in offspring age and the difference in offspring birth mass were fixed effects. The duration help given was marginally significantly influenced by the duration of help received (*β ± SE*: 0.004 ± 0.002, *P* = 0.097), and it was not significantly influenced by relatedness (*β ± SE*: 0.33 ± 0.86, *P* = 0.70), absolute rank difference (*β ± SE*: 0.005 ± 0.009, *P* = 0.59), similarity of offspring sex (*β ± SE*: 0.08 ± 0.09, *P* = 0.39), difference in offspring age (*β ± SE*: -0.01 ± 0.04 *P* = 0.76), and the difference in offspring birth mass (*β ± SE*: -0.11 ± 0.12, *P* = 0.36). We ran a second model that was similar to the first model, however we changed the similarity of offspring sex variable from (1 = same sex, 0 = different sex) to a categorical variable with 4 levels (FF, FM, MM, MF). The duration help given was not significantly influenced by the duration of help received (*β ± SE*: 0.003 ± 0.002, *P* = 0.19), relatedness (*β ± SE*: 0.35 ± 0.86, *P* = 0.69), absolute rank difference (*β ± SE*: 0.004 ± 0.009, *P* = 0.68), difference in offspring age (*β ± SE*: -0.03 ± 0.03 *P* = 0.45), and the difference in offspring birth mass (*β ± SE*: -0.11 ± 0.11, *P* = 0.34). The duration of help given was significantly influenced by the similarity of offspring sex (FM vs FF: *β ± SE*: -0.59 ± 0.24, *P* =0.02, MM vs FF: *β ± SE*: -0.80 ± 0.29, *P* =0.01), and there was a tendency for the duration of help given to be influenced by the comparison between MF vs FF (*β ± SE*: -0.37 ± 0.21, *P* =0.09). The post hoc Tukey comparisons of the similarity of offspring sex for MF vs FM (*β ± SE*: 0.22 ± 0.29, *P* = 0.85), MM vs FM (*β ± SE*: -0.22 ± 0.22, *P* = 0.72) and MM vs MF (*β ± SE*: -0.44 ± 0.24, *P* = 0.22) did not significantly affect the duration of allonursing within dyads.**

**2013 closely-related group. We first ran the model with an interaction between the duration of help received and relatedness, but the interaction was not significant and was removed from the model. The duration help given was the response variable, and we the duration of help received, relatedness, absolute rank difference, similarity of offspring sex (1 = same sex, 0 = different sex), and the difference in offspring birth mass were fixed effects. The difference in offspring age was removed, since its vif value was the highest and was > 20: once removed, all other vif values were below 2.7. The duration help given was not significantly influenced by the duration of help received (*β ± SE*: -0.009 ± 0.009, *P* = 0.34), relatedness (*β ± SE*: -1.05 ± 5.41, *P* = 0.85), absolute rank difference (*β ± SE*: 0.18 ± 0.13, *P* = 0.18), similarity of offspring sex (*β ± SE*: 0.05 ± 0.42, *P* = 0.91), and the difference in offspring birth mass (*β ± SE*: 0.26 ± 0.39, *P* = 0.52). We ran a second model that was similar to the first model, however we changed the similarity of offspring sex variable from (1 = same sex, 0 = different sex) to a categorical variable with 4 levels (FF, FM, MM, MF). The difference in offspring age was removed, since its vif value was the highest and was > 7: once removed, all other vif values were below 2.7. The duration help given was not significantly influenced by the duration of help received (*β ± SE*: -0.007 ± 0.009, *P* = 0.50), relatedness (*β ± SE*: -4.05 ± 6.08, *P* = 0.53), absolute rank difference (*β ± SE*: 0.22 ± 0.14, *P* = 0.17), the difference in offspring birth mass (*β ± SE*: 0.52 ± 0.46, *P* = 0.30), and similarity of offspring sex (FM vs FF: *β ± SE*: -0.03 ± 1.04, *P* = 0.98; MF vs FF: *β ± SE*: -0.45 ± 0.58, *P* = 0.48; MM vs FF: *β ± SE*: -0.67 ± 1.01, *P* = 0.54; post hoc Tukey comparisons MF vs FM: *β ± SE*: -0.42 ± 0.98, *P* = 0.97; post hoc Tukey comparisons MM vs FM: *β ± SE*: -0.65 ± 0.88, *P* = 0.88; post hoc Tukey comparisons MM vs MF: *β ± SE*: -0.22 ± 0.90, *P* = 0.99).**

**2013 distantly-related group. We first ran the model with an interaction between the duration of help received and relatedness, but the interaction was not significant and was removed from the model. The duration help given was the response variable, and we the duration of help received, relatedness, absolute rank difference, similarity of offspring sex (1 = same sex, 0 = different sex), difference in offspring age and the difference in offspring birth mass were fixed effects. The duration help given was not significantly influenced by the duration of help received (*β ± SE*: -0.002 ± 0.009, *P* = 0.86), relatedness (*β ± SE*: 14.31 ± 16.03, *P* = 0.42), absolute rank difference (*β ± SE*: 0.28 ± 0.19, *P* = 0.21), similarity of offspring sex (*β ± SE*: 0.52 ± 0.53, *P* = 0.39), difference in offspring age (*β ± SE*: -0.009 ± 0.047 *P* = 0.86), and the difference in offspring birth mass (*β ± SE*: -0.02 ± 0.29, *P* = 0.95). We ran a second model that was similar to the first model, however we changed the similarity of offspring sex variable from (1 = same sex, 0 = different sex) to a categorical variable with 4 levels (FF, FM, MM, MF). The duration help given was not significantly influenced by the duration of help received (*β ± SE*: 0.0005 ± 0.0182, *P* = 0.98), relatedness (*β ± SE*: 23.85 ± 26.98, *P* = 0.48), absolute rank difference (*β ± SE*: 0.38 ± 0.19, *P* = 0.29), difference in offspring age (*β ± SE*: 0.02 ± 0.08 *P* = 0.84), the difference in offspring birth mass (*β ± SE*: -0.14 ± 0.85, *P* = 0.89), and similarity of offspring sex (FM vs FF: *β ± SE*: -1.56 ± 1.18, *P* =0.32; MF vs FF: *β ± SE*: -1.76 ± 2.11, *P* =0.52; MM vs FF: *β ± SE*: -1.37 ± 1.59, *P* =0.49; post hoc Tukey comparisons MF vs FM: *β ± SE*: -0.19 ± 1.95, *P* = 1.00; post hoc Tukey comparisons MM vs FM: *β ± SE*: 0.20 ± 1.23, *P* = 1.00; post hoc Tukey comparisons MM vs MF: *β ± SE*: 0.39 ± 1.21, *P* = 0.99).**

**We wrote the following on lines 104-106: “****Kin discrimination requires that individuals can discriminate relatives from non-relatives and occurs by the use environmental or genetic cues [34].”**

**There is no experimental study of kin discrimination in reindeer, however Engelhardt et al. (2014) investigated the misdirected parental care hypothesis (i.e. either due to milk-theft or** **mismothering by a lack of efficient kin-recognition mechanisms) proposes that lactating females inadvertently transfer milk to the offspring of other parents (Roulin 2002).** **The non-filial (NF) rate of rejections were significantly greater than the filial (F) rate of rejections (mean difference ± *SE*: 0.242 ± 0.032; Paired *t* test: *t*_(24)_ = 7.644, *P* < 0.001), which supported the milk-theft hypothesis but not the mismothering hypothesis (Engelhardt et al. 2014).**

**In 2012, there were 234 (9.6 %) NF solicitations where the NF offspring arrived first and without other offspring: only 3 (0.1 %) solicitations by NF offspring alone were successful. In addition, there were 3 (0.1 %) solicitations with NF offspring arriving first, successfully allosuckling and the F offspring was beside its mother and suckled second (Engelhardt et al. 2014). In 2013, there were 65 (20.4 %) NF solicitations where the NF offspring arrived first and without other offspring: only 3 (0.9 %) solicitations by NF offspring alone were successful. In 2013, there were no successful solicitations with the NF offspring arriving first and the F offspring arriving second (Engelhardt et al. 2016). In both years, > 99.0% of successful allonursing bouts occurred after the F offspring was suckling. These results suggest that mothers can discriminate their own offspring from the offspring of other mothers, and they suggest that offspring can discriminate their own mother from other lactating females.**

**The following is based on observations rather than recorded data. During the calving seasons and shortly before multiple rut seasons, we observed mothers and their own offspring emitting sounds until they were near each other, i.e. the mothers and offspring ran or walked towards each other. Following this, they stood beside each other. During the calving season, we were able to check if they were mother and offspring based on their collars, and they were all the mother and her offspring. There were no observed mistakes. We did not record this data. Nonetheless, this also suggests that mothers can discriminate their own offspring from the offspring of other mothers, and this suggests that offspring can discriminate their own mother from other lactating females.**

**We wrote the following on lines 143-148: “****There is indirect evidence suggesting that reindeer can discriminate kin. Reindeer mothers rejected offspring attempting to allosuckle more often than they rejected their offspring attempting to suckle [89]. Reindeer mothers rejected nearly all attempts by offspring to allosuckle before the mother’s offspring was suckling [79,89], and most allosuckling attempts by offspring occurred after the mother’s offspring was suckling [89].”**

**R2_11:** 11. “In 2012, all reindeer mothers allonursed 1 or more offspring, and we recorded 1383 allonursing bouts (73). In the closely and distantly, genetically related groups in 2013, we recorded 113 and 48 allonursing bouts, respectively, performed by 8 of the 8 reindeer mothers in the closely-related group and by 7 of the 8 reindeer mothers in the distantly-related groups (72).”

Please provide the average ± 95%CI of the frequency of nursing and allonursing bouts for 2012 and for both the groups of 2013.

1. What was the focal duration for each experimental mother dyad?

2. Was the focal duration held constant across all the experimental dyads within and between years? I do not think so (at least for between years): this is evident from the huge difference in the frequency of allonursing bouts between 2012 and 2013.

3. If not, did the authors correct for inter-dyad variation in focal duration when calculating frequencies of help given and help received?

Please include these in the manuscript (perhaps as a supplementary information).

How many researchers coded the behavioural data? If this was more than one, was there high inter-observer reliability? How was it measured? Please include this in the methods.

**RE_R2_11: We wrote the following on lines 433-438: “The number of nursing bouts per mother was 207.04 ± 7.95 (95% CI: 191.46 – 222.62) in the 2012 group, 49.25 ± 2.48 (95% CI: 44.39 – 54.11) in the closely-related group and 59.38 ± 7.89 (95% CI: 43.91 – 74.84) in the distantly-related group in 2013. The number of allonursing bouts per mother was 55.32 ± 4.02 (95% CI: 47.44 – 63.20) in the 2012 group, 14.13 ± 3.15 (95% CI: 7.95 – 20.30) in the closely-related group and 6.00 ± 2.12 (95% CI: 1.84 – 10.16) in the distantly-related group in 2013.”**

**In 2012, there were 3 observers and 65 observation days over 10 weeks. One observer collected 70 % of the data and observed the reindeer over 10 weeks. There were 2 additional observers. One observer observed the reindeer from May to mid-June, and she was replaced by another observer that took over her place from mid-June to the end of the study. In 2013, there was 1 observer, and both groups were observed for 5 h/day for 25 observation days over 5 weeks.**

**In 2012, there were 3 observers. In 2013, there was 1 observer. We wrote the following on lines 275-278: “****There were 3 observers in 2012 and 1 observer in 2013. Inter-observer reliability was assessed in 2012 between the pairs of observers using Pearson correlation coefficients for the duration of allonursing, and the index of concordance for the identities of mothers and offspring allonursing [103].” We wrote the following on lines 430-433: “The Pearson correlation coefficients for the duration of allonursing between the 3 pairs of observers were 0.997 (N = 418), 0.969 (N = 217), and 0.999 (N = 45). The identities of mothers and offspring allonursing were were reliably measured between the 3 pairs of observers (indexes of concordance = 1.0).”**

**Within year, each reindeer mother has the same observation duration. Within year, mothers only vary based on how often and for how long they nursed and allonursed, not based on observation duration. Nursing and allonursing observations were recorded using behaviour sampling with continuous recording (Martin & Bateson, 2007). The reindeer formed 1 herd in each of the 3 groups, as such there is no variation in focal duration within year. Within year, each female was observed for the same amount of time. Between years, there are large differences due to the number of weeks and hours of observations. Given the difference between years, we ran the analyses separately by group (3 groups: 1 group in 2012 and 2 groups in 2013), as suggested by the reviewer. Those who nursed and/or allonursed more often, were recorded more often, however all mothers within a year had the same observation duration. Thus, there is no need to account for different observation durations, since all mothers within a year had the same observation duration and we ran separate models for each group.**

**R2_12:** 12. “In this study, we revisited the associations between help given and i) help received and ii) pairwise genetic relatedness and their interactions by updating the assessment of reciprocal and kin discriminated allonursing by including all data collected in 2012, i.e. all allonursing bouts lasting > 5 s rather than only the data with exact known duration, and by adding a 2nd year of data, i.e. data collected in 2013.”

This takes me back to the previous-to-previous comment. Have the researchers checked for the effect of relatedness on allonursing duration? It is possible for frequencies and durations to produce different results. Without this knowledge, it is inappropriate to include all the bouts >5 s without weighting them in the models. I suggest the authors to first make sure that frequencies and durations of allonursing bouts follow similar pattern with respect to pairwise relatedness and other predictors. Alternatively, perform two sets of analyses: one with the frequencies and the other with the durations of allonursing bouts and compare the results. Just based on the results of frequencies one cannot conclude the effect of relatedness. Or the conclusion should be such that pariwise genetic relatedness does/does not significantly influence frequency of allonursing in this population of semi-domesticated reindeers.

**RE_R2_12: Please see our reply RE_R2_10.**

**Within year, each reindeer mother has the same observation duration. Within year, mothers only vary based on how often and for how long they nursed and allonursed, not based on observation duration. Nursing and allonursing observations were recorded using behaviour sampling with continuous recording (Martin & Bateson, 2007). The reindeer formed 1 herd in each of the 3 groups, as such there is no variation in focal duration within year. Within year, each female was observed for the same amount of time. Between years, there are large differences due to the number of weeks and hours of observations. Given the difference between years, we ran the analyses separately by group (3 groups: 1 group in 2012 and 2 groups in 2013), as suggested by the reviewer. Those who nursed and/or allonursed more often, were recorded more often, however all mothers within a year had the same observation duration. Thus, there is no need to account for different observation durations, since all mothers within a year had the same observation duration and we ran separate models for each group.**

**R2_13:** 13. “We generated a dominance hierarchy for reindeer mothers in each group using observations of agonistic interactions with a winner and loser identified. We reported the Landau linearity index (86). The dominance hierarchy in the closely related group tended to be linear, with a Landau linearity index of 0.857 (72). The dominance hierarchy in the distantly related group was linear, with a Landau linearity index of 0.988 (72). In 2012, the dominance hierarchy tended to be linear, with a Landau’s index of linearity of 0.785 (70). Ranks were given values ranging 1 to 25 in 2012 and ranging 1 to 8 in both groups in 2013, with larger numbers representing the most dominant mother and 1 representing the least dominant mother.”

Please mention the behaviours that were used to score agonistic interactions (which behaviours, descriptions of behaviours, their frequency, how winners and losers were identified, etc.). If this has been already described in any of the published papers, include references. Please also include the duration of observation for each dyad during which the agonistic interactions were scored. Was this similar across dyads or was it controlled for before constructing the dominance hierarchies?

In 2012, a dominance hierarchy was constructed with all the study individuals regardless of their relatedness. However, in 2013, two separate dominance hierarchies were constructed based on relatedness (i.e., for the two groups). Therefore, the highest absolute rank difference can be 24 in 2012 but only 7 in 2013, which can be also seen in the file Data 1.csv. Is there any evidence to support that these two methods of constructing dominance hierarchies with respect to relatedness would not affect the results? The replicates for rank differences ranging from 8 to 24 will only be from the year 2012, but the replicates for the rank differences ranging from 1-7 will be from both 2012 and 2013. Please bear in mind that these replicates (2012 and 2013) are not similar because of the major difference in the method of constructing the hierarchies.

1. If certain individuals are removed from the larger herd and then the dominance hierarchy is constructed, does the rank relationship between pairs of females remain the same as when they were in the larger herd?

2. If it does not, what do the (temporary) absolute rank differences in the experimental groups mean for the individuals?

3. Do the rank differences in the experimental groups have any biological significance at all?

4. How stable are these dominance ranks? I would think it will take more than the experimental period for the ranks of individuals to become stable, unless, for e.g., if A>B>C is the case and upon removing B, the relationship is still A>C immediately from the time B is removed and continues to be the case if B does not return. If the dominance hierarchy is constructed for the entire herd, would the researchers find this to be the case? Essentially, though the ranks of the experimental animals would themselves differ upon removal from the larger herd, would the hierarchical positions of the experimental animals remain the same before and after removal?

5. Please provide information on when and for how long the dominance data were collected in each year.

**RE_ R2_13: We wrote the following on lines 248-257: “Four behaviour sampling sessions of agonistic interactions of one hour each, using continuous recording, were conducted daily in 2012 [103]. Throughout each observation day in both 2012 and 2013, agonistic interactions were opportunistically scored using ad libitum sampling and continuous recording methods. An agonistic interaction was recorded as resolved when an individual showed a submissive behaviour (“lose”), and the other did not (“win”). Unresolved agonistic interactions were recorded as unresolved when neither animal showed a submissive behaviour. The agonistic interactions scored were displacement, head threat, push, chase, kick, boxing, and other interactions [109,110], and their associated submissive behaviours were scored as ‘flee’ or ‘walk away’ if submission occurred. The rank of female reindeer is fairly stable throughout the year [111], except for a very short time immediately following the shedding of antlers [110–112].”**

**We wrote the following on lines 292-295: “****We recorded 4471 resolved, i.e. a winner and a loser were identified, agonistic interactions between mothers in 2012 [87]. We recorded 517 resolved agonistic interactions between mothers in the closely related group and 627 agonistic interactions between mothers in the distantly-related group in 2013 [79].”**

**Within year, each reindeer mother has the same observation duration. Mothers only vary based on how often they were part of agonistic interacts, not based on observation duration.**

**As the reviewer suggested, we analyzed the data for the decision rules for the 3 groups separately to account for the differences between the groups and between years.**

**The rank of female reindeer is fairly stable throughout the year (Hirotani, 1990), except for a very short time immediately following the shedding of antlers (Espmark, 1971a; Thomson, 1977). We do not have the agonistic data of the entire herd in both years. We cannot inform the reviewer about the rank relationship between pairs of females when they were in the larger herd. Social dominance hierarchies are formed in many animal species, and they influence access to resources, reduce costly fights over resources and dominant individuals often have a greater access to important resources. A difference in social rank in each group of reindeer can impact access to resources and reduce costly fights over resources, e.g. food, allonursing.**

**R2_14:** 14. “To assess the direct reciprocity decision rule and the interaction between the direct reciprocity and kin discrimination decision rules, we ran a generalized linear mixed model with a Poisson distribution with the number of help given as a response variable, and the interaction between the number of help received and the pairwise genetic relatedness, the absolute rank difference, the offspring birth mass difference, the similarity in offspring sex (categorical variable) as fixed effects.”

From R script: m1_givereceive_data_int <-glmer(givencount ~ receivedcount*LRM + ABSOLUTERANKDIFFERENCE + sexsimilarity + birthmassdiff + (1 | id1_factor) + (1 | id2_factor) + (1|year), family="poisson", data=givereceive_data)

summary(m1_givereceive_data_int) # interaction is non-sig

overdisp_fun(m1_givereceive_data_int)

vif(m1_givereceive_data_int)

m1_givereceive_data <-glmer(givencount ~ receivedcount + LRM + ABSOLUTERANKDIFFERENCE + sexsimilarity + birthmassdiff + (1 | id1_factor) + (1 | id2_factor) + (1|year), family="poisson", data=givereceive_data) #,glmerControl(optimizer="bobyqa", optCtrl = list(maxfun = 100000))

summary(m1_givereceive_data)

overdisp_fun(m1_givereceive_data)

vif(m1_givereceive_data)

“The interaction between the number of help received and pairwise genetic relatedness did not significantly affect the number of help given (*β ± SE*: 0.25 ± 0.21, *P* = 0.24), which did not support our prediction of the interaction between the direct reciprocity and kin discrimination decision rules: the interaction was removed from the model.”

Please state how ‘sexsimilarity’ was coded in the model. Was it something similar to FF = -1, FM = 0, MF = 1, MM = 2 or FF = 0 and MM = 1?

What about the factors offspring age difference as well as mothers’ age difference for each dyad? Do they bear any effect on help given? Is there collinearity between female age and female rank? If so, checking age differences between mothers would not be necessary. Otherwise, consider adding these factors to the model. Later run reduced models and perform likelihood ratio tests with all the models to choose the best model/s.

Here, it is the dyad on which the data on help given and received are scored rather than on individual mothers as in the case of generalized reciprocity. Therefore, here dyad identity should be the random factor (as dyads are the replicates) and not individual mothers of the dyad. (i.e., (1 | id1_id2) instead of (1 | id1_factor) + (1 | id2_factor)).

A link function has not been included in the code. For Poisson models, the link function should be log. Please check if R takes link function as log for Poisson models by default. If not, the models will have to be rerun with the appropriate link function.

The model should not be run only with the factor receivedcount*LRM, and the interaction should not be removed just because the *P* value is not significant. The original hypothesis was to check for the effect of the interaction term; therefore, it should be retained regardless of its *P* value. Full model has to be run, and the inferences will have to be made based on the results of the best model. The model should use receivedcount + LRM + receivedcount*LRM.

In the methods section, please write in detail about the model diagnostics that were run – for e.g.,

1. How was independence of residuals tested?

2. How was homogeneity of variance tested?

3. How was model stability tested (DFBeta/DFFit)? If the model is unstable, this reduces inferential power, and the conclusions should be drawn with care.

**RE_R2_14: For all the models in the main text and in the Rscript, there is a section at the end of the Rscript with additional analyses for the reviewer. In these sections of the Rscript, we provided the codes. It is took long to present the model selection by both information theoretic approach (AICc) and likelihood ration test, model validation, influential values, outliers and model results. The response would span hundreds of pages. As a summary, the models do not change the biological significant and interpretation of the results. According to model selection by both the information theoretic approach (AICc) and the likelihood ration tests, the best models include the variables for direct reciprocity and generalized reciprocity (i.e. help received and help given) and for relatedness (pairwise genetic relatedness) without additional variables. Our response to this comment is quite long, because we took the time to show much of the results of several models.**

**In R, the default link function for family= “poisson” is log, thus the model has a link function. The random effects cannot be the dyads, since each dyad is in the data set only once. The identity of the mothers (id1 and id2) are repeated measures, so we use the identity of the mothers as random effects (1| id1) + (1|id2).**

**For models with a poisson distribution, the main concern is overdispersion. When our models are not overdispersed, we keep the distribution. If a model is overdispersed, we first change for a negative binomial distribution and test for overdispersion. If there is still overdispersion in the model with a negative binomial distribution, we then run an observation-level random effects model with a poisson-lognormal distribution (Harrison 2014), which accounts for overdispersion by adding a random effect at the observation level. For a poisson distribution, the homogeneity of variance assumption is violated because as the mean increases the variance also increase (if Y is the observed count, then E(Y) = var(Y) = λ. By definition, the mean of a Poisson random variable must be equal to its variance. We checked for dependence of residuals by plotting the residuals (deviance residuals) against each fixed effect. For generalized linear mixed models with a poisson distribution, we follow Zuur et al.’s (2009) book on mixed effects models in R and the QCBS R workshop (**[**https://r.qcbs.ca/workshops/r-workshop-07/**](https://r.qcbs.ca/workshops/r-workshop-07/)**).**

**With regards to p-hacking, running likelihood ratio tests (as the reviewer suggests, which leads to 55 likelihood ratio tests for each model) or an information theoretic approach is often not much better (Mundry et al. 2011), and assessing the statistical significance of the “best” model is also likely to produce an inflated effect (Forstmeier et al. 2017). We kept our original choices of fixed effects in the revised manuscript to avoid p-hacking and approaches that are often not much better (Mundry et al. 2011; Forstmeier et al. 2017). Based on the reviewer’s comment about likelihood ratio test, we show the results of model selection below. We used 2 methods for model selection, even though the reviewer only asked for the likelihood ratio test. First, we used an information theoretic approach using the package MuMIn to compare the models with different fixed effects and to compare model with the 2 different variables for the similarity of offspring sex (2 levels vs 4 levels). Models with AICc values within 2.0 were considered equivalent. Second, we applied the method that the reviewer suggested and used the lrtest function from the lmtest package to compare models. The models within 2 deltaAICc values did not differ based on the likelihood ratio tests. Based on AICc and likehood ratio tests, we conclude that we could have run the models with only received help and relatedness as fixed effects.**

**With regards to outliers and influential scores, we wrote the following on lines 311-322: “****Following a recommendation by Kline [123], all continuous variables were standardized to z-scores to detect outliers with absolute z-score values greater than 3.3, and we used the scale function to calculate the z-scores. If individuals with outlier scores or influential scores were not from the same population as the rest of the individuals, then it may have been best to remove that case from the sample [123]. We assessed for influential values with dfbetas and Cook’s d. The cutoff values for dfbetas and Cook’s were based on 2/√N and 4/N, respectively, with N representing the number of mothers (2012: N = 25; both groups in 2013: N = 8). The cutoff values for dfbetas were 0.40 in the 2012 group and 0.71 in both of the 2013 gorups, and the cutoff values for Cook’s d were 0.16 in the 2012 group and 0.50 in both of the 2013 groups. The individuals did belong to the population, and it was not best to remove these individuals or values from the sample. The influential values were real data points from the study population, so we kept them to avoid generating biased results due to selective removal of outliers [123–125].” We did not selectively remove outliers, since this practice is generally discouraged because it has a high potential of generating biased results (Holman et al. 2016; Forstmeier et al. 2017). We ran dfbetas and cook’s d. For dfbetas, we considered values as influential when absolute values were greater than 2/√N, and N was the number of individuals in each group (N = 25 in 2012, 8 both groups in 2013). As such dfbetas absolute values greater than 0.4 in 2012 and 0.71 were considered influential. For cook’s d, we considered values as influential when absolute values were greater than 4/N (2012: 0.16, 2013: 0.5). For the reviewer, we ran the models without the influential values. For the reviewer, we ran models to assess the impact of the z-score outliers by changing them to the highest value below an absolute z-score of 3.3, i.e. corrected outlier values (Kline, 2009). As stated in this paragraph, the individuals did belong to the population, and it was not best to remove these individuals from the sample. The outliers were real data points from the study population, so we kept them to avoid generating biased results due to selective removal of outliers (Kline, 2009; Forstmeier et al. 2017; Holman et al. 2016).**

**We initially ran models with the similarity of offspring sex as a categorical variable with 2 levels (1 = same sex, 0 = different sex). This matches with matrix analyses, such as a matrix for a Mantel test or a multiple regression quadratic assignment procedure (Farine and Whitehead 2015). Based on the reviewer’s comment, we also created the similarity of offspring sex as a categorical variable with 4 levels (FF, FM, MM, MF; FF was the level to which all other levels were compared in the model, and we subsequently ran a Post-hoc Tukey comparisons for the additional comparisons). We would prefer keep the similarity of offspring sex variable with 2 levels. There are not many rows of data in both groups in 2013. For example, there is only 1 row for the FF level of offspring similarity in the closely-related group in 2013, so we would prefer to continue to use the variable with 2 levels, which provides more rows in both levels for both groups in 2013. In addition, the models with the similarity of offspring sex with 2 levels return the lowest AICc values, based on an information theoretic approach.**

**Reciprocal interactions can occur between related and unrelated individuals, such that the correlated pay-offs can interact with relatedness (Taborsky et al. 2016), however reciprocal interactions are often not assessed between related individuals. We have predictions to assess i) if reindeer mothers allonurse according to the direct reciprocity decision rule, ii) if reindeer mothers allonurse according to the kin discrimination decision rule, and iii) if reindeer mothers allonurse according to an interaction between the direct reciprocity decision rule and the kin discrimination decision rule (see lines 163-175 in the Introduction section). Our first (original) prediction is not an interaction between help received and relatedness, as the reviewer suggests. We assess if reciprocal interactions interact with relatedness for allonursing in reindeer, and they do not. As such, we remove the interaction term to then test the predictions for the direct reciprocity and kin discrimination decision rules. In a model with an interaction, the first-order variable are conditional main effects, not main effects. As such, writing the model as the reviewer suggests, i.e. receivedcount + LRM + receivedcount*LRM, does not change the model output compared to writing it as we have, i.e. receivedcount*LRM (this is also equivalent to receivedcount + LRM + receivedcount:LRM). As such, we cannot interpret conditional main effects in the same way that we interpret main effects. When a variable interacts with another variable (e.g. receivedcount interacts with LRM), the interpretation of the conditional main effect, e.g. receivedcount, is conditional on the other variable, e.g. LRM, when the other variable is held at zero. Thus, the model with the interaction term assesses prediction 6 (lines 171-173: “To assess if reindeer mothers allonursed according to an interaction between the direct reciprocity and kin discriminaton decision rules, we predicted that 6) the effect of help received on help given should depend on the relatedness between pairs of mothers”), but not prediction 1 (lines 163-165: “To assess if reindeer mothers allonursed according to the direct reciprocity decision rule, we predicted that 1) mothers should give more help to the offspring of mothers who previously helped them more often”) nor prediction 3 (lines 166-168: “To assess if reindeer mothers allonursed according to the kin discrimination decision rule we predicted that 3) mothers should give more help to offspring as the pairwise genetic relatedness of mothers increased”). To assess predictions 1 and 3, we need to run a model with the interaction between receivedcount and LRM, since the interaction between receivedcount and LRM is not significant. We are running model to test the predictions, which is not p-hacking.**

**With regards to outliers and influential scores, we wrote the following on lines 311-322: “Following a recommendation by Kline [123], all continuous variables were standardized to z-scores to detect outliers with absolute z-score values greater than 3.3, and we used the scale function to calculate the z-scores. If individuals with outlier scores or influential scores were not from the same population as the rest of the individuals, then it may have been best to remove that case from the sample [123]. We assessed for influential values with dfbetas and Cook’s d. The cutoff values for dfbetas and Cook’s were based on 2/√N and 4/N, respectively, with N representing the number of mothers (2012: N = 25; both groups in 2013: N = 8). The cutoff values for dfbetas were 0.40 in the 2012 group and 0.71 in both of the 2013 gorups, and the cutoff values for Cook’s d were 0.16 in the 2012 group and 0.50 in both of the 2013 groups. The individuals did belong to the population, and it was not best to remove these individuals or values from the sample. The influential values were real data points from the study population, so we kept them to avoid generating biased results due to selective removal of outliers [123–125].”**

**We wrote the following on lines 323-364: “To assess the predictions for the direct reciprocity decision rule (prediction 1), the kin discrimination decision rule (prediction 3) and the interaction between the direct reciprocity and kin discrimination decision rules (prediction 6), we ran one generalized linear mixed model for each group with a Poisson distribution with the number of help given as a response variable, and the interaction between the number of help received and the pairwise genetic relatedness, the absolute rank difference, the offspring birth mass difference, the similarity in offspring sex (categorical variable with 2 levels: same sexes = 1, different sexes = 0) as fixed effects. The offspring birth mass difference and the similarity in offspring sex were control variables. The individual identities of both mothers in each dyad were used as random effects. If the interaction term between the number of help and pairwise genetic relatedness is not significant (i.e. there is no interaction between the direct reciprocity and kin discrimination decision rules; prediction 6), we remove the interaction term to estimate the main effect of the number of received help to test the prediction for the direct reciprocity decision rule (prediction 1) and the main effect of pairwise genetic relatedness to test the prediction for the kin discrimination decision rule (prediction 3). If the interaction term is not significant, we cannot use the conditional main effects to assess the predictions for the direct reciprocity decision rule (prediction 1) and the kin discrimination decision rule (prediction 3). To assess further assess the relationship between help received and help given, a generalized linear mixed model with a Poisson distribution with the same fixed and random effects was conducted with dyads with a tendency to reciprocate, i.e. 95 dyads with RAFI values equal or greater than 0.50, in the 2012 group with the main effects for the number of help received and for pairwise genetic relatedness. The residuals were not overdispersed in most models, except for the 2013 distantly-related group with the main effects for the number of help received and for pairwise genetic relatedness. To account for overdispersion, a generalized linear mixed model with a negative binomial distribution and the same fixed and random effects was conducted, and the residuals were not overdispersed.** **We tested for multicollinearity using the vif function from the car function. Variation inflation factor values of ≥ 5.00 are interpreted as evidence of multicollinearity [126]. There was no multicollinearity in the models, except for the statistical model for the 2013 distantly-related group with the interaction term between the number of help received and pairwise genetic relatedness (the interaction and the conditional main effect of the number of help received both had variation inflation factor values > 130). By removing the interaction term in this model, there was no multicollinearity. In the 2012 group, there were 4 outliers for the number of help received, and 2 outliers for pairwise genetic relatedness. In the closely-related group in 2013, there was 1 outlier for the number of help received. In the distantly-related group in 2013, there was 1 outlier for the number of help received. There were 3, 1, 0, 3, 1, 4 influential values in the models for the 2012 group with the interaction, for the 2012 group with the main effects for the number of help received and pairwise genetic relatedness, for the 2012 group’s dyads with a tendency to reciprocate (RAFI ≥ 0.50) with the main effects for the number of help received and pairwise genetic relatedness, for the 2013 closely-related group with the interaction, for the 2013 closely-related group with the main effects for the number of help received and pairwise genetic relatedness, for the 2013 distantly-related with the main effects for the number of help received and pairwise genetic relatedness, respectively. Model results with the corrected outlier values were similar to the model results with the outliers, so we reported the model results with the outliers. We reported incidence rate ratios, their 95% confidence intervals and conditional R^2^.”**

**Please read the results section from lines 453-538 for the results of the main effects and the interaction.**

**Analyses for the reviewer:**

**We ran correlations between age of mothers and rank of mothers separately for the 3 groups (25 mothers in 2012, and 8 mothers in both groups in 2013). Age of mothers and rank of mothers were moderately correlated in the closely-related group (correlation coefficient: 0.58, N = 8) and the distantly related group (correlation coefficient: 0.59, N = 8), however they were slightly correlated in the 2012 group (correlation coefficient: 0.29, N = 25). Given these values, we did not include age of mothers in the models. However, the absolute age difference of mothers and the absolute rank difference in the dyadic data were not weakly correlated with values of 0.07, 0.08, and 0.02 in the 2012 group, the closely-related group in 2013 and the distantly-related group in 2013. Thus, we included both the** **absolute age difference of mothers and the absolute rank difference. We also added the offspring age difference for each dyad to the analyses. We checked collinearity of the variables and for multicollinearity.**

**2012 group with the interaction term. Based on an information theoretic approach, the model that include the interaction between received count and relatedness is equivalent to the models that additionally include the similarity of offspring sex (both the variable with 2 levels and the variable with 4 levels), however all other models with additional fixed effects have deltaAICc values > 2.0 (see below). The model with the lowest AICc includes the interaction term between received help and relatedness, and the similarity of offspring sex with 2 levels (AICc = 1047.47, df = 7, weight = 0.24), followed by a similar model but with the similarity of offspring sex with 4 levels (deltaAICc = 0.25, df = 9, weight = 0.21). The next model equivalent to the 2 previous models only includes the interaction between received help and relatedness (deltaAICc = 0.26, df = 6, weight = 0.21). Fifty-five models were compared using likelihood ratio tests. Four likelihood ratio tests were significant (p < 0.05), and none of the models with deltaAICc values less than or equal 2.0 differed. We only show the likelihood ratio tests for the comparison of the 3 top models based on AICc. We do not show the results of all possible models to save space in the rebuttal, but we included them in the Rscript). We also provide the results of the model with similarity of offspring sex with 4 levels. For the assessment of the prediction of an interaction between received count and relatedness, the variable similarity of offspring sex with 2 or 4 levels did not influence the model results: the interaction between received count and relatedness is not significant. We also provide the results of the model that included absolute age difference of mothers, the absolute rank difference and offspring age difference, and these fixed effects did not significantly influence help given: they were not retained in the model.**

**2012 group with the interaction term. Based on an information theoretic approach, the model that include the interaction between received count and relatedness is equivalent to the models that additionally include the similarity of offspring sex (both the variable with 2 levels and the variable with 4 levels), however all other models with additional fixed effects have deltaAICc values > 2.0 (see below). The model with the lowest AICc includes the interaction term between received help and relatedness, and the similarity of offspring sex with 2 levels (AICc = 1047.47, df = 7, weight = 0.24), followed by a similar model but with the similarity of offspring sex with 4 levels (deltaAICc = 0.25, df = 9, weight = 0.21). The next model equivalent to the 2 previous models only includes the interaction between received help and relatedness (deltaAICc = 0.26, df = 6, weight = 0.21). There were 2 influential dfbetas value, and 1 of these 2 values was an influential cook’s d value. We presented the model results when the influential values were removed, and it did not change the interpretation of the results for the interaction between received count and relatedness. We presented the model results when the outlier z-scores were corrected, and there is a tendency for a significant effect of the interaction when the outlier values are changed, however pairwise genetic relatedness was estimated and received count was observed. We will not correct the values of these 2 variables, since they are real data points from the population. We presented the results of the other 2 models with deltaAICc within 2.0, and we presented the results of the model with the additional variable that the reviewer asked about: the interaction of receivedcount:LRM was not significant.**

**Model results of the best fitting model**

**Plots of residuals vs fixed effects**

**Model without influential values**

**Model account for outliers based on z-scores**

**Models results when offspring sex similiraty has 4 levels instead of 2 levels with deltaAICc within 2.0 and weight of 0.21. The interaction receivedcount:LRM is not significant.**

**Model results with deltaAICc within 2.0 and weight of 0.21. The interaction receivedcount:LRM is not significant.**

**Model results when absolute rank difference of mothers, absolute age difference of mothers and absolute age difference of offspring are included**

**2012 group for the main effects of received help and relatedness. Based on an information theoretic approach, the model that includes the main effects of received help and relatedness with the similarity of offspring sex is equivalent with 3 additional models, however all other models with additional fixed effects have deltaAICc values > 2.0 (see below). The model with the lowest AICc includes the main effects between received help and relatedness, and the similarity of offspring sex with 2 levels (AICc = 1046.96, df = 6, weight = 0.25), followed by a similar model but without the similarity of offspring sex (deltaAICc = 0.26, df = 5, weight = 0.22). The next model equivalent to the 2 previous models includes the main effects of received help and relatedness with the similarity of offspring sex is equivalent with 4 additional models (deltaAICc = 0.34, df = 8, weight = 0.21). The next model equivalent to the 3 previous models includes the main effects of received help and relatedness with the similarity of offspring sex is equivalent with 2 additional models and the absolute rank difference (deltaAICc = 2.0, df = 7, weight = 0.09). There was 2 influential values. We presented the model results when the influential values were removed, and it did not change the interpretation of the results for the received help and relatedness. We presented the model results when the outlier z-scores were corrected, and result interpretation did not differ from the results without correcting the outliers. We presented the results of the other 3 models with deltaAICc within 2.0, and we presented the results of the model with the additional variable that the reviewer asked about: the received help and relatedness were not significant.**

**Model results of the best fitting model. Received help is significant and significant even after an alpha correction of 0.05/2 for the 2 models. Relatedness is not significant.**

**Plots of residuals vs fixed effects**

**Model without influential values. Received help is significant. Relatedness is not significant.**

**Model account for outliers based on z-scores. Received help is significant. Relatedness is not significant.**

**Model results with deltaAICc within 0.26 and weight of 0.22. Received help is significant and significant even after an alpha correction of 0.05/2 for the 2 models. Relatedness is not significant.**

**Models results when offspring sex similiraty has 4 levels instead of 2 levels with deltaAICc within 0.34 and weight of 0.21. Received help is significant. Relatedness is not significant.**

**Model results with deltaAICc within 2.0 and weight of 0.09. Received help is significant and significant even after an alpha correction of 0.05/2 for the 2 models. Relatedness is not significant.**

**Model results when absolute rank difference of mothers, absolute age difference of mothers and absolute age difference of offspring are included and none were significant. Received help is significant and significant even after an alpha correction of 0.05/2 for the 2 models. Relatedness is not significant.**

**2013 closely-related group with the interaction term. Based on an information theoretic approach, the model that include the interaction between received count and relatedness is the best model (AICc = 115.74, weight = 0.75) and there are no equivalent models with a deltaAICc within 2.0. The next 3 models included the similarity of offspring sex with 2 levels, and the best model with the similarity of offspring sex with 4 levels had a deltaAICc of 10.00 and a weight of 0.01. There were 5 influential dfbetas valus. We presented the model results when the influential values were removed, and it did not change the interpretation of the results for the interaction between received help and relatedness. We presented the model results when the outlier z-scores were corrected, and it did not change the interpretation of the results for the interaction between received help and relatedness. We presented the results of the model with the additional variable that the reviewer asked about: the interaction of receivedcount:LRM was not significant.**

**Model results of the best fitting model. Received help and relatedness are not significant.**

**Plots of residuals vs fixed effects**

**Model without influential values. Received help and relatedness are not significant.**

**Model account for outliers based on z-scores. Received help and relatedness are not significant.**

**Model results when absolute rank difference of mothers, absolute age difference of mothers and absolute age difference of offspring are included: none are signficant. Received help and relatedness are not significant.**

**2013 closely-related group for the main effects of received help and relatedness. Based on an information theoretic approach, the model that include the interaction between received count and relatedness is the best model (AICc = 112.40, df = 5, weight = 0.70) and there are no equivalent models with a deltaAICc within 2.0. The next 3 models included the similarity of offspring sex with 2 levels, and the best model with the similarity of offspring sex with 4 levels had a deltaAICc of 8.34 and a weight of 0.01. There were 2 influential dfbetas value, and 1 of these 2 values was an influential cook’s d value. We presented the model results when the influential values were removed, and it did not change the interpretation of the results for the received help and relatedness. We presented the model results when the outlier z-scores were corrected, and it did not change the interpretation of the results for the received help and relatedness. We presented the results of the model with the additional variable that the reviewer asked about: the received help and relatedness were not significant.**

**Model results of the best fitting model. Received help and relatedness are not significant.**

**Plots of residuals vs fixed effects**

**Model without influential values. Received help and relatedness are not significant.**

**Model account for outliers based on z-scores. Received help and relatedness are not significant.**

**Model results when absolute rank difference of mothers, absolute age difference of mothers and absolute age difference of offspring are included: none are signficant. Received help and relatedness are not significant.**

**2013 distantly-related group with the interaction term. Based on an information theoretic approach, the model that include the interaction between received count and relatedness is the best model (AICc = 84.44, df = 7, weight = 0.96) and there are no equivalent models with a deltaAICc within 2.0. The next 3 models included the similarity of offspring sex with 2 levels, and the best model with the similarity of offspring sex with 4 levels had a deltaAICc of 28.99 and a weight of 0.00. There was 1 influential dfbetas value, and there were 2 influential cook’s d values. We presented the model results when the influential values were removed, and it did not change the interpretation of the results for the interaction between received help and relatedness. We presented the model results when the outlier z-scores were corrected, and it did not change the interpretation of the results for the interaction between received help and relatedness. We presented the results of the model with the additional variables that the reviewer asked about: the interaction of receivedcount:LRM was not significant. There is multicollinearity in all the models presented below, however we cannot remove the fixed effects in the best fitting model in order to test the predictions.**

**Model results of the best fitting model. Received help and relatedness are not significant.**

**Plots of residuals vs fixed effects**

**Model without influential values. Received help and relatedness are not significant.**

**Model account for outliers based on z-scores. Received help and relatedness are not significant.**

**Model results when absolute rank difference of mothers, absolute age difference of mothers and absolute age difference of offspring are included. Absolute difference of age of mothers is significant, however this model has a deltaAICc of 41.07 and weight of 0.00. Received help and relatedness are not significant. The model is overdispersed, and there is multicollinearity.**

**2013 distantly-related group for the main effects of received help and relatedness. Based on an information theoretic approach, the model that include the interaction between received count and relatedness is the best model (AICc = 79.20, df = 6, weight = 0.90) and there are no equivalent models with a deltaAICc within 2.0. The next 3 models included the similarity of offspring sex with 2 levels, and the best model with the similarity of offspring sex with 4 levels had a deltaAICc of 22.13 and a weight of 0.00. There was 1 influential dfbetas value, which was the same 1 influential cook’s d value. We presented the model results when the influential values were removed, and it did not change the interpretation of the results for the interaction between received count and relatedness. We presented the model results when the outlier z-scores were corrected, and it did not change the interpretation of the results for the interaction between received count and relatedness. We presented the results of the model with the additional variable that the reviewer asked about: the received help and relatedness were not significant.**

**Model results of the best fitting model. Received help and relatedness are not significant.**

**Plots of residuals vs fixed effects**

**Model without influential values. Received help and relatedness are not significant.**

**Model account for outliers based on z-scores. Received help and relatedness are not significant.**

**Model results when absolute rank difference of mothers, absolute age difference of mothers and absolute age difference of offspring are included. Received help and relatedness are not significant. Absolute age difference of mothers is significant, however this model had a deltaAICc of 28.19 and a weight of 0.00.**

**R2_15:** 15. “There were 5 outliers for the number of help received, 2 outliers for pairwise genetic relatedness, and 1 outlier for offspring birth mass difference with an absolute z-score values greater than 3.3. The outliers were real data points, so we kept them. To assess the impact of these outliers, we changed them to the highest value below an absolute z-score of 3.3, and we ran the model with these corrected outlier values.”

How was this done? Which function was used? Please write these clearly in the methods sections and provide references for this method. Without going through the data and the R code, it will be impossible for anyone to understand or replicate this step. DFBeta (mentioned above) is a good measure to detect influential cases.

**RE_R2_15: We used the scale function, which is in base R, to generate the z-score values. Please see RE_R2_14 for our response.**

**R2_16:** 16. “Observations of successful allonursing across bouts were summed within dyads for the closely and distantly, genetically related groups. To assess dyadic reciprocity, we used the reciprocity index presented by Mitani (84), which has been used to assess dyadic reciprocity for allogrooming (84,85) and for allonursing (70), to create the reciprocal allonursing frequency index (RAFI).

Where *aAB* is the amount of allonursing that individual *A* gave to individual *B*’s offspring, *aBA* is the amount of allonursing that individual *B* gave to individual *A*’s offspring, and *aA + aB* is the total amount of allonursing between the two individuals.”

If I have understood this correctly, only the successful allonursing bouts were considered to calculate RAFI. It is then not meaningful to run the below model:

**RE_R2_16: The index of reciprocity, RAFI, is based on successful allonursing bouts, and it is a measure of direct reciprocity. Nonetheless, mothers in dyads with higher RAFI values could reject the other mother’s offspring of mothers more often or less often, which would affect the likelihood of allonursing. As such, the likelihood of allonursing and its effect size, the odds ratio, should be greater as RAFI values increase. We argue that this is a good way to further assess that mother are reciprocal, and we do not agree that the likelihood model is not meaningful. The combination of the model for the direct reciprocity decision rule with help given as the response variable and the likelihood to allonurse model strengthen the results.**

**Please see lines 365-384 in the methods and lines 553-619 in the results.**

**R2_17:** 17. “To further assess the direct reciprocity decision rule and the interaction between the direct reciprocity and the kin discrimination decision rules, we ran a generalized linear mixed model with a binomial distribution with successful (1) and unsuccessful (0) allonursing attempts as a response variable, and RAFI, pairwise genetic relatedness, offspring sex (categorical variable), and absolute rank difference. The response variable of the likelihood of allonursing model equals 0 when an attempt is unsuccessful, i.e., the lactating female did not allonurse the offspring of another mother, and equals 1 when an attempt is successful, i.e., the lactating female did allonurse the offspring of another mother.”

This is because higher the RAFI higher would be the likelihood of successful allonursing.

1. It would be better to design this model as:

glmer (RAFI ~ pairwise genetic relatedness + absolute rank difference + similarity in offspring sex + id1_id2 age difference + offspring1_offspring2 age difference +

offspring1_offspring2 birth mass difference + (1 | id1_id2) + (1 | year), family = “X”, link = “x”, data = data)

through which it is possible to understand if genetic relatedness, rank difference, etc., has any effect on the extent of direct reciprocity. Here again reduced models would have to be run and the full and the reduced models should be compared with likelihood ratio tests. Variance Inflation Factor should also be measured for the model.

Here again, upon going through the R script, dyad identity should be the random factor and not the identities of the individual mothers.

2. Alternatively, Mantel tests can be performed with the pairwise RAFI and genetic relatedness matrices separately for each year.

**RE_R2_17: Dyads cannot be the random factor: please see our response in RE_R2_14 (paragraph 6). RAFI is count-proportional data.**

**We ran the suggested model. We cannot model it with a beta distribution, since the RAFI data contains zeros and ones. We multiplied the RAFI values by 100 and rounded them to the nearest interger in order to run analyses. A value of 50 is equivalently comparable to a value of 0.50. This opens the possibility for Poisson and negative binomial distributions. We attempted a zero-inflated Poisson, but the models did not converge, i.e. the parameter values are estimated. With a Poisson distribution, the model converges the residuals are not overdispersed, and there is no multicollinearity.**

**We wrote the following in the methods section on lines 385-397: “****To further assess the prediction for the kin discrimination decision rule (prediction 5), we ran separate generalized linear mixed models per study group with either a Poisson distribution or a negative binomial distribution, if the residuals were overdispersed, with the direct reciprocity index, RAFI, as the response variable. The values of RAFI were multiplied by 100 and rounded to the nearest integer. The fixed effects were the pairwise genetic relatedness, the absolute rank difference of mothers, the difference in offspring birth mass, and the absolute difference in the age of mothers. The individual identities of both individuals in each dyad were used as random intercept effects. In the 2012 data, there were 2 outliers for pairwise genetic relatedness with an absolute z-score values greater than 3.3. There were no outliers in the 2013 data. All values were real data points from the population, so we kept them. To assess the impact of these outliers, we changed them to the highest value below an absolute z-score of 3.3, and we ran the model with these corrected outlier values. Model results with the corrected outlier values were similar to the model results with the outliers, so we reported the model results with the outliers.** **There was no multicollinearity.” Please read the results on lines 620-646.**

**We kept the likelihood model from R2_16: see our response to RE_R2_16.**

**R2_18:** 18. “For each model, when the interaction between pairwise genetic relatedness and either i) the number of help received or ii) RAFI was not significant, the interaction was removed from the model.”

As explained above, individual factors should not be removed post hoc based on *P* value. Complete models will have to be run unless there is a strong reason to not do so.

“To assess the generalized reciprocity decision rule, we asked if receiving help in general increased subsequent help given, and we ran a general linear model with a negative binomial distribution with the overall number of help given by each reindeer mother as a response variable, and the overall number of help received by each reindeer mother as an independent variable.”

From R script: m2_givereceive_gen_rep <-glm.nb(givencount ~ receivedcount, data=givereceive_gen_recip)

1. Please state the reason for using a negative binomial distribution (overdispersion); include the function used and the reference.

2. It is important to add mother_ID (as there are repeated observations on the same individuals) and year as random factors (if same method was followed for both the years) – i.e., (1 | mother_ID), (1 | year).

3. Factors such as mother’s age, mother’s dominance rank, offspring sex, relatedness to the receiver should be included as control fixed factors, and VIF should be measured.

4. If the focal duration is not the same across different mothers, please include offset.

**RE_R2_18: With regards to the removal of the interaction term for the predictions for the direct reciprocity and kin discrimination decision rules, please see our response RE_R2_14 paragraph 6 for a full explanation of our predictions and why we have to remove the non-significant interaction term to assess the predictions 1 and 3. However, there is no removal of an interaction term for the model assessing if reindeer mothers help according to the generalized reciprocity decision rule. We revised the model and included individual and year as random intercept effects.**

**The generalized reciprocity decision rule states help anyone if helped by someone. The cognitive demands for generalized reciprocity only require the ability to remember whether or not one received help without having to identify or remember the partner(s) (Taborsky et al. 2016). The identity of the individual from whom one received or did not receive help is not irrelevant and so is the identity of future partners. Generalized reciprocity may be a very important decision rule, because it there are fewer cognitive demands than the direct reciprocity decision rule. We are assessing if reindeer mothers allonurse according to the generalized reciprocity decision rule, i.e. help anyone if helped by someone. Gerald Carter assessed if vampire bats shared food according to the generalized reciprocity decision rule using a bivariate regression between help given and help received (**[**https://socailbat.org/2013/03/24/generalized-reciprocity-in-vampire-bats/**](https://socailbat.org/2013/03/24/generalized-reciprocity-in-vampire-bats/)**). Michael Taborsky assessed if Norway rats give help to partners according to the generalized reciprocity decision rule using experimental designs with cooperators and defectors (Rutte and Taborsky 2007). The goal of this prediction is to assess the decision rule, i.e. help anyone if helped by someone, not the variables about the partners. This is not a decision based on i) the relatedness between partners, ii) the age of mothers, iii) the sex of offspring of the mothers.**

**We ran generalized linear mixed models with a Poisson distribution (log link function) with the given help as the response variable and the received help as the fixed effect with an offset for the number of observation days (65 in 2012 and 25 in 2013). The identity of the mothers and the years were random intercept effects. The residuals were not overdispersed.**

**For the reviewer, we used an information theoretic approach to compare this model to the models with different combinations of age of mother and offspring sex included, and the model we ran had the lower AICc value and the highest weight (AICc = 354.65, weight = 0.5). The models with offspring sex and with both age of mothers and offspring sex had AICc values > 2 and had weights of 0.17 and 0.08, respectively. Help received significantly influenced help given (*β ± SE*: 0.0038 ± 0.0018, *P* =0.034, conditional R^2^ = 0.290) in the model with the lowest AICc (AICc = 354.65, weight = 0.5). Help received significantly influenced help given (*β ± SE*: 0.0038 ± 0.0018, *P* =0.03, conditional R^2^ = 0.282) when age of mothers was included (deltaAICc = 1.4, weight = 0.25). Help received marginally significantly influenced help given (*β ± SE*: 0.0034 ± 0.0018, *P* =0.061, conditional R^2^ = 0.283) when offspring sex was included (deltaAICc = 2.16, weight = 0.17). Help received marginally significantly influenced help given (*β ± SE*: 0.0034 ± 0.0018, *P* =0.058, conditional R^2^ = 0.290) when both age of mothers and offspring sex was included (deltaAICc = 3.59, weight = 0.08).**

**We wrote the following on lines 398-409: “****To assess the prediction for the generalized reciprocity decision rule (prediction 8), we assessed if receiving help in general increased subsequent help given, and we ran a general linear model with a Poisson distribution with the overall number of help given by each reindeer mother as a response variable, and the overall number of help received by each reindeer mother as an independent variable. To account for the different number of observation days between years, an offset for the number of observation days was included. There were 2 random intercept effects: the identity of mothers receiving and giving help and year were a random intercept effects. There were two influential values and one outlier. The generalized reciprocity decision rule states help anyone if helped by someone. The cognitive demands for generalized reciprocity only require the ability to remember whether or not one received help without having to identify or remember the partner(s) [20,21]. The identity of the individual from whom one received or did not receive help is not irrelevant and so is the identity of future partners.”**

**We wrote the following in the results section on lines 649-653: “****The overall number of help given increased as the overall number of help received increased (*β ± SE*: 0.004 ± 0.002, *P* = 0.033, IRR (95% CI): 1.0038 (1.0004 – 1.0077)). The intercept was significant (*β ± SE*: -0.78 ± 0.27, *P* < 0.001). The random intercept effect for the mother explained 0.29 (SD = 0.54) of the variance, and the random intercept effect for the year explained 0.12 (SD = 0.34) of the variance. The model’s conditional R^2^ was equal to 0.29.”**

**In addition, the reviewer will find in the Rscript that we ran the generalized reciprocity model with offspring sex and the age of mothers. We ran model selection with both an information theoretic approach and multiple likelihood ratio tests. For all the models in the main text and in the Rscript, there is a section at the end of the Rscript with additional analyses for the reviewer. In these sections of the Rscript, we provided the codes. It is took long to present the model selection by both information theoretic approach (AICc) and likelihood ration test, model validation, influential values, outliers and model results. The response would span hundreds of pages. As a summary, the models do not change the biological significant and interpretation of the results.**

**R2_19:** 19. “To compare direct reciprocity and generalized reciprocity, we ran a generalized linear mixed model with a Gaussian distribution with the latency to give help after receiving help in days as a response variable, the comparison between the direct reciprocity and generalized reciprocity decision rules as levels of a categorical fixed effect, and the mother receiving help, the mother giving help and year as random intercept effects.”

From R script: mDRorGRlatencyyears <- lmer(log(latency_day) ~ DRorGR + (1|year) + (1|CALFSMOTHER) + (1|DOEID), data = latency_grvsdr)

1. Since this is from the perspective of the mother that has received help and is subsequently offering help, I do not think it is necessary to include (1|DOEID) – i.e., the receiver, as a random factor.

2. It is important to add confounding variables in the model: Do mothers with male offspring allonurse as frequently as mothers with a female offspring (offspring sex)? Do older mothers allonurse as frequently as young mothers (mother’s age)? Does the presence of closely related individuals influence the latency period (presence of kin – yes/no)? etc.

**RE_R2_19: We wrote the following on lines 409-425: “To compare the latency to give help to anyone after having receiving help (prediction 9), we calculated the observed latencies to give help to anyone after having received help. When the time at the end of a received help was not recorded, we added the mean duration of allonursing bouts, i.e. 15 s, to the time at the start of receiving help. This decision has no significant effect on the latencies to give help to anyone after having received help, since reindeer mothers i) did not simultaneously help each other, ii) rarely helped each other within the same day, and the latencies between help received and help given typically span multiple days****.** **It is not possible to assign each allonursing bout given after having received help as direct reciprocity or generalized, so we calculated the latencies to give help after receiving help according to both direct reciprocity and generalized reciprocity. To compare direct reciprocity and generalized reciprocity, we ran a generalized linear mixed model with a Gaussian distribution with the latency to give help after receiving help in days as a response variable. The comparison between the direct reciprocity and generalized reciprocity decision rules as levels of a categorical variable, the age of mothers and offspring sex (M vs F), and pairwise genetic relatedness were the fixed effects. There were two random intercept effects, i.e. the identity of mothers receiving help and giving help in return and year were random intercept effects. The latency to give help after receiving help in days was log-transformed, so the model residuals were normally distributed.”**

**We wrote the following on lines 653-663: “****The latency to give help after receiving help was shorter for generalized reciprocity than direct reciprocity by 18.31 days (mean ± SE latency for direct reciprocity: 19.42 ± 0.45 days (range: 1.00 – 64.09 days); mean ± SE latency for generalized reciprocity: 1.11 ± 0.04 days (range: 4.63e-5 – 16.31 days); model *β ± SE*: -3.68 ± 0.07, *P* < 0.001, Fig 3). As the age of the mother increased, the latency to give help after receiving help decreased (*β ± SE*: -0.17 ± 0.07, *P* = 0.017). Sex of the offspring (M vs F: *β ± SE*: 0.09 ± 0.14, *P* = 0.51) and pairwise genetic relatedness (*β ± SE*: 0.13 ± 0.62, *P* = 0.83) did not significantly influenced the latency to give help after receiving help. The intercept was significant (*β ± SE*: 2.52 ± 0.25, *P* = 0.01). The random intercept effect for the mothers explained 0.08 (SD = 0.29) of the variance, and the random intercept effect for the groups explained 0.13 (SD = 0.36) of the variance. The model’s conditional R^2^ was equal to 0.56.”**

**R2_20:** 20. “For all models, there was no multicollinearity.”

Please elaborate on which function was used to test for multicollinearity and provide the relevant stats and references.

It is difficult to comment on the results, as there are serious issues with data collection and statistical analyses.

**RE_R2_20: We wrote the following on lines 346-352: “We tested for multicollinearity using the vif function from the car function. Variation inflation factor values of ≥ 5.00 are interpreted as evidence of multicollinearity [126]. There was no multicollinearity in the models, except for the statistical model for the 2013 distantly-related group with the interaction term between the number of help received and pairwise genetic relatedness (the interaction and the conditional main effect of the number of help received both had variation inflation factor values > 130). By removing the interaction term in this model, there was no multicollinearity.” There was no multicollinearity in all other models.**

**R2_21:** 21. “The interaction between the number of help received and pairwise genetic relatedness did not significantly affect the number of help given (*β ± SE*: 0.25 ± 0.21, *P* = 0.24), which did not support our prediction of the interaction between the direct reciprocity and kin discrimination decision rules: the interaction was removed from the model.”

As explained before, this is not acceptable, and potentially amounts to *P*-hacking.

**With regards to the removal of the interaction term for the predictions for the direct reciprocity and kin discrimination decision rules, please see our response RE_R2_14 paragraph 6 for a full explanation of our predictions and why we have to remove the non-significant interaction term to assess the predictions 1 and 3.**

**R2_22:** 22. “The number of help given increased as the number of help received increased (*β ± SE*: 0.05 ± 0.02, *P* = 0.009, Fig 1A), which supported our prediction that reindeer mothers allonursed according to the direct reciprocity decision rule.”

Looking at the graph, it seems that the model is underfitting the data. I would suggest the researchers to focus on and report effect sizes and not only *P* values. What was the *R*2 for this and all the models? Potentially, the estimated coefficient could also be a measure of the effect size. Going by this, what does a 0.05 increase in help received mean? How biologically significant is it?

Please include units in the graphs and elsewhere. For e.g., in Figure 1 A, given and received allonursing bouts per and in Figure 1B, given allonursing bouts per?

**RE_R2_22: The conditional R^2^ are estimated with either the performance package’s r2 function to return the conditional R^2^ for generalized linear mixed models by Nakagawa or the MuMIn package’s r.squaredGLMM function. The conditional R^2^ for the models reported in RE_R2_14 are as follows. For the 2012 group with the interaction between help received and relatedness, conditional R^2^ = 0.524. For the 2012 group with the main effects for help received and relatedness, conditional R^2^ = 0.528. For the 2013 closely-related group with the interaction between help received and relatedness, conditional R^2^ = 0.438. For the 2013 closely-related group with the main effects for help received and relatedness, conditional R^2^ = 0.413. For the 2013 distantly-related group with the interaction between help received and relatedness, conditional R^2^ = 0.438. For the 2013 distantly-related group with the main effects for help received and relatedness, the conditional R^2^ = 0.36, based on the delta method of the r.squaredGLMM function in the MuMIN package. For this model, the condition R^2^ in the performance package cannot be estimated. This may be due to the observation-level random-effect. For the 2013 distantly-related group with the main effects for help received and relatedness, R^2^ = 0.179 (Nagelkerke’s R^2^) since both random effects account for 0 variance.**

**The conditional R^2^ of the generalized reciprocity model in RE_R2_18 is 0.290.**

**The link function for Poisson distrition is log. A Possion coefficient is the difference between the logs of expected counts. For a 1 unit change in the predictor variable, the difference in the logs of expected counts is expected to change by the coefficient, given that the other predictor variables in the model are held constant. The inverse function for natural logarithm (log in R) is to exponentiate. The exponentiated coefficients are also known as the incidence rate ratios (IRR). We also provide the 95% confidence intervals for the IRR. For the 2012 group with the main effects for help received and relatedness, the estimated coefficient equals 0.05. The IRR for help received is 1.05 (95% CI: 1.01 – 1.09). I provide 3 ways to interpret the estimated coefficient for help received of 0.05. If a reindeer mother were to receive 1 additional help, the rate ratio for help given would be expected to increase by a factor of 1.05. Each additional 1 help received is associated with an increase in help given by a factor of 1.05. In other words, a reindeer mother who receives 2 bouts of help is expected to give help, on average, 1.05 times more than a reindeer mother who received 1 bout of help. Each additional help received is associated with 5% more help given.**

**We wrote the following on lines 467-471: “The number of help given increased as the number of help received increased (main effect: *β ± SE*: 0.05 ± 0.02, *P* = 0.0096, IRR (95% CI): 1.05 (1.01 – 1.09), Fig 1A) in the 2012 group. For a one unit increase in the number of help received, the number help given is expected to increase by a factor of 1.05 (95% CI for IRR: 1.01 – 1.09). If a mother received help one, 10 or 15 times, the mother is expected to give help 2.61, 4.10 and 5.26 times, respectively.” We included the IRR and their 95% CI in the main text. The readers could easily have exponentiate the coefficients to calculate the IRR and its 95% confidence intervals.**

**For the likelihood to allonurse models, we included the odds ratio (OR) and their 95% CI in the main text.**

**We added the data points in the figures.**

**R2_23:** 23. “The number of help given was not significantly affected by the pairwise genetic relatedness (*β ± SE*: 0.48 ± 0.60, *P* = 0.42, 290 Fig 1B), which did not support our prediction that reindeer mothers allonursed according to the kin discrimination decision rule.”

Here again, report effect size, and it seems that the model is underfitting the data based on the graph. Additionally, why is the predictor range so small (pairwise genetic relatedness: -0.1 to 0.2)? How can a general inference about genetic relatedness be made from this limited range? That “reindeer mothers do not allonurse according to kin discrimination decision rule” is an inferential overreach.

**RE_R2_23: We wrote the following in the results section on lines 471-473: “****The number of help given was not significantly influenced by the pairwise genetic relatedness (main effect: *β ± SE*: 0.39 ± 0.61, *P* = 0.52, IRR (95% CI): 1.48 (0.44 – 4.89), Fig 1B).” We reported the incidence rate ratio with its 95% confidence interval.**

**The Kutuharju Field Reindeer Research Station population is the largest reindeer field station in the world. Nonetheless, the herd is managed to avoid inbreeding. Reindeer herders in Lapland exchange reindeer within their cooperative in order to avoid inbreeding.**

**We assessed if reindeer mothers allonursed according to the kin discrimination decision rule. Yet, it is unlikely that a larger range of pairwise genetic relatedness would be positively associated help given or the index of reciprocity (RAFI), since reindeer mothers and yearling daughters tend to associate throughout the year, except during calving when their associations do not differ from a control sample of unrelated females (Hirotani 1990). Within the range of observed pairwise genetic relatedness, pairwise genetic relatedness did not significantly influence the number of help given nor the likelihood of allonursing, which is supported by Hirotani’s (1990) findings. In addition, RAFI decreased as pairwise genetic relatedness increased. For empirical studies in taxa with a high social complexity, i.e. eusocial and cooperative breeding social systems, the association between relatedness and cooperation was negative [67–69] or neutral [70–76]. Several studies that experimentally manipulated relatedness found that relatedness reduced cooperation rather than increase it [42,69,79,80], which differs with kin selection theory.**

**We wrote the following on lines 711-714: “A larger range of relatedness is unlikely to positively associate with help given, the likelihood of allonursing and RAFI, since reindeer mothers rarely associate with related individuals during the calving season and during the rest of the year [111,139], except for mothers and their yearling adult daughters during the mating season.”**

**We added the following in the methods on lines 188: “****The herd is managed to avoid inbreeding.”**

**We added the following in the discussion on lines 694-711: “Relatedness did not positively associate with help given in the 3 groups, which provided correlational evidence that did not support the prediction for the kin discrimination decision rule (prediction 3: main effect of relatedness on help given). Two additional results further validated that reindeer mothers did not help each other by allonursing according to the kin discrimination decision rule. Relatedness did not positively associate with the likelihood of allonursing in the 3 groups, which provided correlational evidence that did not support the second prediction for the kin discrimination decision rule (prediction 4: main effect of relatedness on the likelihood of allonursing). The neutral association between relatedness and RAFI in the 2012 group and the negative association between relatedness and RAFI in the 2013 groups did not support the third prediction for the kin discrimination decision rule (prediction 5: main effect of relatedness on RAFI). These results suggest that reindeer mothers did not cooperate according to the kin discrimination decision rule, i.e. preferentially help kin. There are also neutral associations between relatedness and cooperation in correlational studies of eusocial and cooperative breeding social systems [70–76]. The negative association between RAFI and relatedness suggests that relatedness reduces cooperation during the calving season in reindeer. A negative association between relatedness and cooperation is supported by i) correlational studies of eusocial and cooperative breeding social systems [67–69], ii) a comparative analysis of eusocial hymenoptera [78], experimental manipulations of relatedness [42,69,79,80], and a theoretical model [81].”**

**R2_24:** 24. “The interaction between RAFI and pairwise genetic relatedness did not significantly affect the likelihood to allonurse (*β ± SE*: -0.43 ± 2.11, *P* = 0.84), which did not support our prediction of the interaction between the direct reciprocity and kin discrimination decision rules: the interaction was removed from the model. The likelihood of allonursing increased as RAFI increased (*β ± SE*: 0.57 ± 0.14, *P* < 0.001, Fig 2A), which supported our prediction that reindeer mothers allonursed according to the direct reciprocity decision rule.”

As explained before, this analysis needs to be modified. Figure 2A is not meaningful. Always show graphs with the data and the fitted model.

**RE_R2_R24: We ran the suggested model with RAFI as the response variable, and we kept the likelihood of allonursing model: please see our responses RE_R2_16, RE_R2_17. We disagree with the reviewer that the likelihood to allonurse model is no meaningful (see RE_R2_16). We added the data to all figures.**

**R2_25:** 25. “The likelihood of allonursing was not significantly affected by the pairwise genetic relatedness (*β ± SE*: 0.38 ± 0.78, *P* = 0.62, Fig 2B), which did not support our prediction that reindeer mothers allonursed according to the kin discrimination decision rule.”

This is inferential overreach. We only know (if we assume the model to be appropriate) that within the range of -0.1 to 0.2 pairwise genetic relatedness, relatedness does not have a significant effect on likelihood of allonursing.

**RE_R2_25: We removed “which did not support our prediction that reindeer mothers allonursed according to the kin discrimination decision rule”. We wrote the following on lines 554-556: “The likelihood of allonursing was not significantly affected by the pairwise genetic relatedness (main effect: *β ± SE*: 0.24 ± 0.81, *P* = 0.77, OR (95% CI): 1.27 (0.26 – 6.21), Fig 2B).”**

**Please see our response RE_R2_23 for relatedness.**

**R2_26:** 26. “The overall number of help given increased as the overall number of help received increased (*β ± SE*: 0.011 ± 0.004, *P* = 0.006, Fig 3), which supported our prediction that reindeer mothers allonursed according to the generalized reciprocity decision rule. The latency to give help after receiving help was shorter for generalized reciprocity than direct reciprocity by 18.30 days (mean ± SE latency for direct reciprocity: 19.42 ± 0.45 days (range: 1.00 – 64.09 days); mean ± SE latency for generalized reciprocity: 1.11 ± 0.04 days (range: 0.000046 – 16.31 days); model *β ± SE*: -3.67 ± 0.07, *P* < 0.001, Fig 4).”

These are interesting results, but one can only comment on their soundness after the analysis is properly redone. Please include units in the graphs to help interpret the coefficients.

The authors have to take much care in writing the discussion section. The sentences in the introduction cannot be reproduced as such in the discussion. For e.g.,

From Discussion: The direct reciprocity decision rule explains reciprocal cooperation across several taxa, e.g. mammals (11,21–28) (e.g. vampire bats (22,23) and Norway rats (24–27)), birds (3,4), fishes (19,20), and microorganisms (30,31).

From Introduction: Direct reciprocity was reported across several taxa, including mammals (11,21–28) (e.g. vampire bats, *Desmodus rotundus* (22,23) and Norway rats, *Rattus norvegicus* (24–27,29)), birds (3,4), fishes (19,20), and microorganisms (30,31).

Please explain what has been found in the current study and how it relates to the existing knowledge regarding allonursing in reindeers in both wild and semi-domesticated populations. Please discuss if and how domestication influences the nature of allonursing. Discuss how the species’ social organization and structure can influence allonursing (expectations for each hypothesis will have to be written out clearly) in the introduction, and whether the findings are in line with the expectation should be discussed. Importantly, please discuss all the limitations of the study and how they can be addressed in the future. Please discuss if and how the present study furthers what we know about allonursing, and more generally, cooperation in reindeer societies. Please also discuss if and how the current study contributes to our general knowledge about allonursing. Please do not discuss support for or against hypotheses for which data were not specifically collected and/or that were not formally tested (for e.g., the milk evacuation hypothesis). There are previous works on generalized reciprocity, therefore, I recommend the authors to cite those works (for e.g., Bohn *et al*. 2009: Pup guarding by greater spear-nosed bats) and discuss them. After rework with a larger dataset if there is still support for generalized reciprocity, please discuss why other studies do not find support for it and what factors could have contributed to the lack of evidence and what factors could have led to generalized reciprocity in the study population.

**RE_R2_26: We revised our analyses. We also provide the Rscript for model selection by both an information theoretic approach (AICc) and likelihood ratio tests. We provide the effect sizes and their 95% confidence intervals, and we include the data in all figures. We removed sentences that were reproduced in the discussion. We extensively revised the discussion section according to your suggestions, and most of the discussion was rewritten.**

**Previous studies of allonursing in reindeer reported its occurrence without testing hypotheses to explain allonursing. No variables were tested in these studies, so there was no knowledge about the effect of variables on allonursing in wild and semi-domesticated reindeer populations. Please see our response RE_R2_8. We wrote in the discussion the following on lines 771-780: “****Reindeer offspring suckled 3 times/hr [102] or 1.7 times/hr [104] during the first week, and the frequency of suckling decreased throughout the lactation period. Researchers have reported allonursing in reindeer [98,102,104]. Espmark [102] recorded 85 allonursing bouts, where 14 of the 15 mothers allonursed, and all offspring solicitated allosuckling. Six out of six mothers allonursed (290 allonursing bouts observed), and each of their offspring were allonursed [98]. The hypothesized causes of allosuckling and allonursing were not tested in these studies.** **Allonursing occurs more often in larger groups [6]. Allonursing incidence is likely high in wild reindeer and caribou, since they form very large herds. Sámi reindeer herders observe allonursing in large herds of semi-domesticated reindeer. Cooperation in reindeer may include babysitting, when a mother rests with the offspring while the other mothers forage, however we seldom observed this behaviour.”**

**The two major limitations are i) the number of observation days in 2013, and ii) 24 h monitoring of reindeer. The latency to return help received according to the direct reciprocity decision rule is on average 19.42 observation days. With 25 observation days in 2013, the study period is too short to fully capture the signal of the direct reciprocity decision rule. Nonetheless, we should point out that that 2013 study was designed to manipulate relatedness at the extremes of pairwise genetic relatedness in the population.** **Twenty-four hour monitoring of the reindeer mothers would increase the number of observed allonursing bouts and rejections, and the signal for direct reciprocity may be detected at a much shorter period than an average of 19.42 observation days.**

**We wrote the following on lines 684-693: “The question of “****The question of “why was there no correlational evidence of reindeer mothers in both 2013 groups cooperating with social partners according to the direct reciprocity decision rule?” is unlikely to be based on a different evolved explanation for cooperation in communal breeding. The difference between years is the time period of observation, i.e. 65 observation days in 2012 versus 25 observation days in 2013. The latency to given help after receiving help according to the direct reciprocity decision rule for allonursing reindeer mothers is on average 19.42 ± 0.45 days, yet the observation period spans 25 observation days in 2013. Returning allonursing help received was not observed to occur simultaneous, within a few minutes and within a day because i) lactation is the most energetically costly aspect of mammalian biology [8,9], and ii) reindeer have smaller udders than most ungulates [97].”**

**We discussed additional limitations in this study on lines 781-791: “Reindeer are neither diurnal nor nocturnal, and their activity is not limited by daylight during the calving season. In the Arctic, there is daylight 24 h/day during the calving season. Nursing and allonursing in reindeer occur over 24 h/day based on preliminary observations. Twenty-four hour monitoring of the reindeer mothers would increase the number of observed allonursing bouts and rejections, and the latency to return help received according to the direct reciprocity may be detected at a much shorter period than an average of 19.42 observation days. Such monitoring may also increase the effect size for the association between help received and help given according to the generalized reciprocity decision rules. Another limitation of this study is that we did not study the entire research population during the calving season. Future research of allonursing in reindeer should assess this behaviour in the context of the species’ social organization and structure to identify additional variables that influence allonursing in reindeer.”**

**We included the study by Bohn et al. (2019) on lines 749-753: “The generalized reciprocity decision rule has been assessed in the greater spear-nosed bats (*Phyllostomus hastatus*) [145]. In the greater spear-nosed bats, alloparental care is not explained by kinship and mistaken identify, and it is unlikely to be explained by direct reciprocity and generalized reciprocity [145]. An interplay between cooperative foraging and group membership may explain alloparental care in greater spear-nosed bats [145].”**
