# Supplementary material for: Evidence suggesting that reindeer mothers allonurse according to the direct reciprocity and generalized reciprocity decision rules
Source: PLoS One. 2023 Dec 14;18(12):e0295497. doi: 10.1371/journal.pone.0295497 (PMC10721201; doi:10.1371/journal.pone.0295497)
Supplement: Attachment — Submitted filename: Response to Reviewers.docx [file pone.0295497.s015.docx]

PONE-D-22-28248R2

Evidence suggesting that reindeer mothers allonurse according to the direct reciprocity and generalized reciprocity decision rules

PLOS ONE

Dear Dr. Engelhardt,

Thank you for submitting your manuscript to PLOS ONE. After careful consideration, we feel that it has merit but does not fully meet PLOS ONE’s publication criteria as it currently stands. Therefore, we invite you to submit a revised version of the manuscript that addresses the points raised during the review process.

I’ve just taken over as Editor on this submission because the previous Editor was unable to continue. Both Reviewers have now recommended Acceptance (with very minor changes), but on my reading of the manuscript, there are a couple of issues that need to be addressed before publication. I don’t want to ask for substantial changes at this late stage, but since one reviewer is demanding some minor changes anyway, you should attend to the following points in your revision.

AE_1: First, the methods makes it sound like the reindeer were removed from their mothers, or that the mother-infant pairs were removed from the rest of the herd. If so, this would remove any contextual cues of who their kin were. Many mammals rely on such contextual cues like “who did I grow up with”, “who else does my mother care for”, so removing the animals would remove their ability to distinguish kin from non-kin. By analogy, it would be like removing a human infant (or non-verbal mother-infant pair) from their family, then testing many years later whether that child would be more generous towards its siblings without telling it who the siblings were. Back to this manuscript, the authors need to clarify in the manuscript who was removed and when. If the allonursers were removed when they themselves were young, then they would have had no contextual cues of kinship, so it is unsurprising that they didn’t allonurse more for kin. In this case, the authors would need to tone down all their conclusions about kinship, discuss how the reindeer didn’t have any contextual cues to help them discriminate kin, starting right from the abstract with a statement like “though this is possibly because they lacked contextual kinship cues”. If instead it was the allonurser’s offspring who were removed, then the authors need to discuss the consequences of this. For example, would it result in more allnursing than normal, given that the females didn’t have their own offspring to invest in?

**Re_AE_1: Thank you for this comment. This is a misunderstanding, and we revised the text to help clarify. This study is from the point of the view of the mothers, and we investigated if the mothers allonursed according to three different decision rules. To help clarify, we revised the Methods section on lines 228-234: “No mother was removed from their own mother when they themselves were young. Mothers were not separated from their offspring. Thus, reindeer mothers’ ability to distinguish kin from non-kin was not affected. Mothers gave birth to their offspring and raised their off-spring with the rest of the herd in the calving paddocks for two to 5 weeks. Mother-offspring pairs selected for the study were then separated from the rest of the herd, which was re-leased in a large calving ground area. The study animals and the herd were rejoined at the end of the study.”**

AE_2: Second, in the discussion, it sounds like the 2013 meta-analysis is underpowered. Normally, I’d recommend that you conduct an internal meta-analysis of the 2012 and 2013 data to check the overall effects – this would be the best way to report these data. However, given how late it is in the review process, I’m not going to demand such a major overhaul. Instead, you should do one of the following: a) do a quick power analysis to demonstrate that 2013 does indeed have sufficient power; b) acknowledge everywhere (including up front & in the abstract) that the 2013 null results could be due to insufficient power; c) do the internal meta-analysis of 2012 & 2013 I suggested and adjust the discussion accordingly (this would improve the presentation of the results).

**Re_AE_2: Thank you for bringing up this important point. As the editor suggested, we ran two meta-analyses, and both were significant. We ran one meta-analysis with the incidence rate ratio as the effect size based on the generalized linear mixed models with a Poisson distribution for the association between the amount of help given and received. We ran a second meta-analysis with the odds ratio as the effect size based on the generalized linear mixed models with a binomial distribution with the likelihood of allonursing as the response variable.**

**We wrote the following on lines 422-428: “****Two internal meta-analyses were performed to assess the direct reciprocity decision rule among the three groups of reindeer mothers with the inverse variance method of pooling to assess the pooled effect size with fixed effects models based on the between-study heterogeneity. The effect sizes were the incidence rate ratio and the odds ratio, and both effect sizes were derived from the generalized linear mixed models. The estimates of the effect sizes were comparable among groups, since they were generated from generalized linear mixed models with the same distributions, and fixed and random effects.”**

**We wrote the following in the Results section on lines 696-711: “****Meta-analyses for the direct reciprocity decision rule**

**For each additional help received by reindeer mothers, the rate of help given increased by 1.05 (fixed effects model for the incidence rate ratio: pooled effect size = 1.05 (95% CI: 1.01 – 1.08), p < 0.001, N = 3 effect sizes), which supports the direct reciprocity decision rule for the amount of help given and received. All effect sizes shared the same true effect size (Q = 2.00, df = 2, p = 0.37; tau^2^ = 0.0001 (95% CI: 0.0000 – 0.2460); I^2^ = 0.1% (95% CI: 0.0% – 89.6%); H = 1.00 (95% CI: 1.00 – 3.10)). The effect size for the 2012 group accounted for 89.7% of the weight for the pooled effect size for the incidence rate ratio, whereas the 2013 closely-related and distantly-related groups accounted for 7.3% and 3.0%, respectively, of the weight for the pooled effect size. The odds of allonursing bout increased as RAFI increased (fixed effects model for OR: pooled effect size = 1.81 (95% CI: 1.38 – 2.39), p < 0.001, N = 3 effect sizes), which supports the direct reciprocity decision rule for the likelihood of a successful allonursing bout. All effect sizes shared the same true effect size (Q = 5.32, df = 2, p = 0.07; tau^2^ = 0.27 (95% CI: 0.00 – 12.36); I^2^ = 62.4% (95% CI: 0.0% – 89.3%); H = 1.63 (95% CI: 1.00 – 3.05)). The effect size for the 2012 group accounted for 87.0% of the weight for the pooled effect size for the OR, whereas the 2013 closely-related and distantly-related groups accounted for 10.1% and 2.8%, respectively, of the weight for the pooled effect size.”**

**We wrote the following in the Discussion section on lines 748-752: “The internal meta-analysis results further supported the direct reciprocity decision rule among the three groups of reindeer mothers in 2012 and 2013, since 1) the rate of help given increased as help received increased, and 2) the odds of allonursing increased as RAFI increased among the 3 groups. The weight of the 2012 group for the pooled effect sizes was ≥ 87.0, which explains the significant internal meta-analysis results among the three groups.”**

**With the addition, we re-read the Discussion several times. We think that the following paragraph remains important for Discussion about the lack of a positive association in the 2013 groups on lines 756-771: “****The question of “why was there no correlational evidence of reindeer mothers in both 2013 groups cooperating with social partners according to the direct reciprocity decision rule?” is unlikely to be explained by the cooperation mechanism of i) by-product mutualism [25,31–34], which cannot be cheated, since allonursing can be cheated by offspring stealing milk [7,108,171], and ii) enforcement [40,41], since neither the mothers nor the offspring coerced the other to transfer milk from the mother to the offspring. Allonursing can be a product of offspring stealing milk [6,7], and reindeer offspring steal milk [108]. Thus, milk parasitism as a form of cooperation by deception plays a role in this communal breeding social system. Nonetheless, additional observation days were very likely required to assess the direct reciprocity decision rule in 2013. The difference between years is the time period of observation, i.e. 65 observation days in 2012 versus 25 observation days in 2013.** **The latency to give help after receiving help according to the direct reciprocity decision rule for allonursing reindeer mothers is on average 19.42 ± 0.45 days, yet the observation period spans 25 observation days in 2013. Returning allonursing help received was not observed to occur simultaneously, within a few minutes and within a day because i) lactation is the most energetically costly aspect of mammalian biology [8,9], and ii) reindeer have smaller udders than most ungulates [121].”**

**The effect sizes for each group were already reported with the model results. We added the codes to the Rscript.**

AE_3: Third, the first few pages of the manuscript are challenging to read. The sentences within the paragraphs seem to jump around in a disconnected way. Many paragraphs are also very long (>1 page), and contain multiple topics that would be better separated into different paragraphs. This isn’t the strongest way to start, and will lose some readers. Ideally, you would think hard about the information in those paragraphs and reorganize the information into smaller packages. However, this late in the review process, I’ll settle for just breaking up the long paragraphs into smaller paragraphs – aim for no more than ½ to ¾ page, though shorter is fine too. Doing so will improve your paper’s readability, and thus its impact.

**Re_AE_3: Thank you for this comment. We revised the introduction and separated paragraphs that contained multiple topics.**

**Comment for the Academic Editor: Please note that the track changes on lines 532-534 with the field code changes and the Spanish (latin america) formatting are odd to me. I keep trying to reject these, and Word keeps bringing this up again and again. I assume this is an issue with the Word/Mendeley plug-in. Please ignore these track changes.**

Once you have addressed these concerns and the final concerns of the one reviewer, then the paper should be acceptable for publication.

Please submit your revised manuscript by Nov 19 2023 11:59PM. If you will need more time than this to complete your revisions, please reply to this message or contact the journal office at [plosone@plos.org](mailto:plosone@plos.org). When you're ready to submit your revision, log on to <https://www.editorialmanager.com/pone/> and select the 'Submissions Needing Revision' folder to locate your manuscript file.

Please include the following items when submitting your revised manuscript:

- A rebuttal letter that responds to each point raised by the academic editor and reviewer(s). You should upload this letter as a separate file labeled 'Response to Reviewers'.
- A marked-up copy of your manuscript that highlights changes made to the original version. You should upload this as a separate file labeled 'Revised Manuscript with Track Changes'.
- An unmarked version of your revised paper without tracked changes. You should upload this as a separate file labeled 'Manuscript'.

If you would like to make changes to your financial disclosure, please include your updated statement in your cover letter. Guidelines for resubmitting your figure files are available below the reviewer comments at the end of this letter.

If applicable, we recommend that you deposit your laboratory protocols in protocols.io to enhance the reproducibility of your results. Protocols.io assigns your protocol its own identifier (DOI) so that it can be cited independently in the future. For instructions see: <https://journals.plos.org/plosone/s/submission-guidelines#loc-laboratory-protocols>. Additionally, PLOS ONE offers an option for publishing peer-reviewed Lab Protocol articles, which describe protocols hosted on protocols.io. Read more information on sharing protocols at <https://plos.org/protocols?utm_medium=editorial-email&utm_source=authorletters&utm_campaign=protocols>.

We look forward to receiving your revised manuscript.

Kind regards,

Pat Barclay
Academic Editor
PLOS ONE

Journal Requirements:

Please review your reference list to ensure that it is complete and correct. If you have cited papers that have been retracted, please include the rationale for doing so in the manuscript text, or remove these references and replace them with relevant current references. Any changes to the reference list should be mentioned in the rebuttal letter that accompanies your revised manuscript. If you need to cite a retracted article, indicate the article’s retracted status in the References list and also include a citation and full reference for the retraction notice.

**Please note that we added two references, which were recently accepted. One is already published (Engelhardt and Taborsky 2023), and the other paper is in the proofs stage (Engelhardt and Taborsky Accepted in Ethology). We added the reference that reviewer 3 commented on, i.e. Stieger et al. 2017, Behav Ecol Sociolbiol 71: 182. We removed McPherson et al. (2001) and Schürch et al. (2010) from the references: they were not cited. We removed two additional citations and moved the order of two citations. These changes affected the numbering of the references and the citations.**

[Note: HTML markup is below. Please do not edit.]

Reviewers' comments:

Reviewer's Responses to Questions

**Comments to the Author**

1. If the authors have adequately addressed your comments raised in a previous round of review and you feel that this manuscript is now acceptable for publication, you may indicate that here to bypass the “Comments to the Author” section, enter your conflict of interest statement in the “Confidential to Editor” section, and submit your "Accept" recommendation.

Reviewer #1: All comments have been addressed

Reviewer #3: (No Response)

2. Is the manuscript technically sound, and do the data support the conclusions?

The manuscript must describe a technically sound piece of scientific research with data that supports the conclusions. Experiments must have been conducted rigorously, with appropriate controls, replication, and sample sizes. The conclusions must be drawn appropriately based on the data presented.

Reviewer #1: Partly

Reviewer #3: Yes

3. Has the statistical analysis been performed appropriately and rigorously?

Reviewer #1: Yes

Reviewer #3: Yes

4. Have the authors made all data underlying the findings in their manuscript fully available?

The [PLOS Data policy](http://www.plosone.org/static/policies.action#sharing) requires authors to make all data underlying the findings described in their manuscript fully available without restriction, with rare exception (please refer to the Data Availability Statement in the manuscript PDF file). The data should be provided as part of the manuscript or its supporting information, or deposited to a public repository. For example, in addition to summary statistics, the data points behind means, medians and variance measures should be available. If there are restrictions on publicly sharing data—e.g. participant privacy or use of data from a third party—those must be specified.

Reviewer #1: Yes

Reviewer #3: (No Response)

5. Is the manuscript presented in an intelligible fashion and written in standard English?

PLOS ONE does not copyedit accepted manuscripts, so the language in submitted articles must be clear, correct, and unambiguous. Any typographical or grammatical errors should be corrected at revision, so please note any specific errors here.

Reviewer #1: Yes

Reviewer #3: Yes

6. Review Comments to the Author

Please use the space provided to explain your answers to the questions above. You may also include additional comments for the author, including concerns about dual publication, research ethics, or publication ethics. (Please upload your review as an attachment if it exceeds 20,000 characters)

Reviewer #1: (No Response)

**Thank you for your comments and effort.**

Reviewer #3: I commend the authors for revising their manuscript satisfactorily. Most of problems I have indicated have been removed. There are just a few issues left as outlined in my detailed comments below.

**Thank you for your comments.**

Detailed comments (by line numbers):

R3_1: 39-40: I think this needs to be amended otherwise it is not clear whether it refers to direct or generalised reciprocity (e.g. by saying something like: “i) increased as the number of help received FROM THE SAME PARTNER increased”

**Re_R3_1: We revised the sentence to include “from the same partner” on lines 39-42: “The number of help given i) increased as the number of help received from the same partner increased in the 2012 group but not in both 2013 groups, ii) was not influenced by relatedness, and iii) was not influenced by an interaction between the number of help received from the same partner and relatedness.”**

R3_2: 41-42: Before the sentence “The overall number of help given increased as the overall number of help received increased” I would add “iv)”.

**Re_R3_2: We added “iv)” before the sentence on lines 42-43: “iv) The overall number of help given increased as the overall number of help received increased.”**

R3_3: 195-200 (line number given in response letter): I’m afraid this is a misunderstanding. In my previous comment “direct reciprocity should generate a higher propensity to provide help than generalised reciprocity; it is less prone to cheating” I meant that DIRECT reciprocity is less prone to cheating, because the behaviour of interacting partners is directly controlled among them by their mutual responses; some researchers therefore allude to reciprocity as being “enforced”. In generalised reciprocity cheating is not directly prevented by punishment but rather indirectly causes a decline in mutual cooperation among interacting partners in a community. Hence I personally would switch the argument around and write something like: “… since DIRECT reciprocity is less prone to cheating than GENERALISED reciprocity. Therefore, EFFECTS OF RECEIVED HELP SHOULD VANISH MORE QUICKLY IF GENERALISED RECIPROCITY APPLIES, SO we predicted that 9) the DISTRIBUTION OF latencIES to give help after receiving help should PEAK AT shorter TIME INTERVALS for GENERALISED reciprocity than FOR DIRECT reciprocity.”

**Re_R3_3: We revised the sentences as suggested on lines 200-205: “****We propose that the propensity to help should be greater according to direct reciprocity than according to generalized reciprocity, since direct reciprocity is less prone to cheating than generalized reciprocity. Therefore, the effects of received help should vanish more quickly if generalized reciprocity applies, so we predicted that 9) the distribution of latencies to give help after receiving help should peak at shorter time intervals for generalized reciprocity than for direct reciprocity.”**

R3_4: 709-712 (line number given in response letter): With my previous comment on this sentence I wanted to point out that also ALLOGROOMING in Norway rats showed “a positive association between help given and help received” (Stieger et al. 2017, Behav Ecol Sociolbiol 71: 182).

**Re_R3_4: We added Stieger et al. (2017) for allogrooming in Norway rats on lines 743-745: “A positive association between help given and help received within dyads is a commonly accepted result for allogrooming by direct reciprocity in primates [62,142,165] and in Norway rats [166] …”**

R3_5: 781-783 (line number given in response letter): Here you should first stress that older mothers respond quicker to received help by returning it after shorter latency periods, and then you should offer potential explanations (e.g. that older mothers are more experienced in reciprocal interactions than younger individuals). What you now state is just a more elaborated description of the result, not an interpretation.

**Re_R3_5: We revised the sentence as follows on lines 821-823: “Older mothers returned received help sooner than younger mothers, and we suggest that older mothers may be more experienced in reciprocal interactions than younger mothers.”**

R3_6: 813: For clarity I propose to write “and reindeer offspring DO steal milk”

**Re_R3_6: We added “do” on lines 761-762: “and reindeer offspring do steal milk [108].”**

R3_7: 819-820: “… not observed to occur simultaneousLY"

**Re_R3_7: We corrected this on lines 768-769: “… not observed to occur simultaneously".**

Michael Taborsky
(I always sign my reviews because I am in favour of transparency in science; cf. Ethology 113 (2007), 1–8)

7. PLOS authors have the option to publish the peer review history of their article ([what does this mean?](https://journals.plos.org/plosone/s/editorial-and-peer-review-process#loc-peer-review-history)). If published, this will include your full peer review and any attached files.


If you choose “no”, your identity will remain anonymous but your review may still be made public.


**Do you want your identity to be public for this peer review?** For information about this choice, including consent withdrawal, please see our [Privacy Policy](https://www.plos.org/privacy-policy).

Reviewer #1: No

Reviewer #3: **Yes:** Michael Taborsky

[NOTE: If reviewer comments were submitted as an attachment file, they will be attached to this email and accessible via the submission site. Please log into your account, locate the manuscript record, and check for the action link "View Attachments". If this link does not appear, there are no attachment files.]

While revising your submission, please upload your figure files to the Preflight Analysis and Conversion Engine (PACE) digital diagnostic tool, <https://pacev2.apexcovantage.com/>. PACE helps ensure that figures meet PLOS requirements. To use PACE, you must first register as a user. Registration is free. Then, login and navigate to the UPLOAD tab, where you will find detailed instructions on how to use the tool. If you encounter any issues or have any questions when using PACE, please email PLOS at [figures@plos.org](mailto:figures@plos.org). Please note that Supporting Information files do not need this step.

*In compliance with data protection regulations, you may request that we remove your personal registration details at any time.* [*(Remove my information/details)*](https://www.editorialmanager.com/pone/login.asp?a=r)*. Please contact the publication office if you have any questions.*
